# The relationship between adiposity and cognitive function: a bidirectional Mendelian randomization study in UK Biobank

**DOI:** 10.1093/ije/dyad043

**Published:** 2023-04-08

**Authors:** Tom Norris, Antoine Salzmann, Albert Henry, Victoria Garfield, Snehal M Pinto Pereira

**Affiliations:** Division of Surgery and Interventional Science, Institute of Sport, Exercise and Health, University College London, London, UK; MRC Unit for Lifelong Health and Ageing at UCL, Institute of Cardiovascular Science, University College London, London, UK; Institute of Cardiovascular Science, University College London, London, UK; Institute of Health Informatics, University College London, London, UK; MRC Unit for Lifelong Health and Ageing at UCL, Institute of Cardiovascular Science, University College London, London, UK; Division of Surgery and Interventional Science, Institute of Sport, Exercise and Health, University College London, London, UK

**Keywords:** Cognition, adiposity, Mendelian randomization, bidirectional, cohort

## Abstract

**Background:**

There may be a bidirectional relationship between cognition and adiposity, whereby poor cognition leads to increased adiposity and vice versa. We aimed to determine whether these findings are causal, by undertaking a bidirectional Mendelian randomization (MR) study.

**Methods:**

A total of 378 877 UK Biobank participants had three adiposity indicators [body fat percentage (BF%), body mass index (BMI) and waist-hip ratio] and two cognitive function measures (reaction time, visual memory). We examined observational associations between each adiposity indicator and cognitive function and vice versa. Using bidirectional inverse-variance weighted MR, we estimated the strength of the adiposity-cognitive function association using genetic instruments for adiposity indicators as our exposures, and we repeated this in the opposite direction using instruments for cognitive function.

**Results:**

In the direction adiposity to cognitive function, MR analyses were generally directionally consistent with observational findings, but all confidence intervals contained the null. In the opposite direction, MR estimates for all adiposity measures on reaction time were imprecise and directionally inconsistent. MR estimates for the effects of visual memory on all adiposity measures indicated worse visual memory was associated with lower adiposity. For example, a 1-unit worse visual memory score was associated with a 1.32% [β = −1.32; 95% confidence interval (CI): −0.77,−1.88] and 3.57% (β = −3.64; 95% CI: −1.84,−5.15) lower absolute body fat percentage and relative body mass index, respectively.

**Conclusions:**

Observational associations of adiposity on cognitive function are likely not causal. In the reverse direction, our consistent findings that worse visual memory is associated with three adiposity indicators provide support for a causal link between worse visual memory and lower adiposity.

Key Messages

- In this pseudo two-sample Mendelian randomization study using genetic instruments from large-scale genome-wide association studies and individual-level data from UK Biobank, we observed no evidence for a causal effect of adiposity on cognitive function.
- In the other direction, there was consistent evidence showing that a worse visual memory resulted in lower body fat percentage (BF%), waist-hip ratio (WHR) and body mass index (BMI).
- Observational associations of adiposity on cognitive function are likely not to be causal. In the reverse direction, we provide support for a causal link between visual memory and adiposity.

## Introduction

The prevalences of obesity (defined as a BMI >30 kg/m^2^) and cognitive impairment are high: globally, 15.7% of females and 11.6% of males are obese^1^ and 6–12% of adults have a mild cognitive impairment (MCI).^2^ The prevalence of obesity and MCI increases with age^3–6^ and, against a backdrop of an ageing population,^7^ their health and economic burdens are likely to continue rising.

In adulthood, obesity has been consistently associated with lower cognitive function,^8^^,^^9^ notably with poor executive function,^10^ intellectual functioning, psychomotor performance and speed, and visual construction.^11^ However, as studies primarily employ BMI as a measure of total adiposity,^10^ the role of adiposity location (i.e. central vs peripheral) in the adiposity-cognition relationship remains uncertain. Some studies have investigated the relationship between indicators of central adiposity [e.g. waist-hip ratio (WHR) and waist circumference (WC)] and cognition, with inconsistent results.^12–17^ Additionally, in terms of total body fatness, studies investigating the association between body fat percentage (BF%) and cognitive function have also provided conflicting evidence.^17–19^

Lower cognitive function has also been associated independently with adiposity.^20–25^ As such, a bidirectional causal relationship may exist whereby lower cognitive function causes increased adiposity and conversely, adiposity causes lower cognitive function.^10^

Mendelian randomization (MR), specifically bidirectional MR, is a strategy that may help unpick the extent to which the pathways between adiposity and cognitive function represent a bidirectional causal pathway. Hagenaars and colleagues^26^ used a bidirectional MR analysis to explore the BMI-‘cognitive ability’ (verbal-numerical reasoning) relationship and found no causal effect in either direction. A limitation of their study was a lack of published genetic variants for cognitive ability at the time of publication. Therefore, single nucleotide polymorphisms (SNPs) for educational attainment were employed as a cognitive ability proxy. Additionally, the use of BMI as a proxy for total adiposity did not permit an investigation into whether specific adiposity indicators were differentially associated with cognitive function. Recently, Wang and colleagues^27^ performed a bidirectional MR of BMI and WHR (adjusted for BMI; WHR_adj_BMI) on cognitive performance and vice versa. They observed conflicting findings in both directions, e.g. in the direction of cognition to adiposity, there was robust evidence that higher cognitive performance caused lower BMI but little evidence for an effect on WHR_adj_BMI. In the reverse direction, there was no effect of BMI on cognitive performance but some evidence for a detrimental effect of higher WHR_adj_BMI. The study predominantly used a single indicator (verbal-numerical reasoning) to represent cognitive performance and thus it is not known how other indicators of cognitive performance may relate to adiposity. Moreover, MR findings in relation to WHR_adj_BMI may be biased and should be avoided.^28^ In light of recent findings that SNPs associated with specific distributions of adiposity are differentially associated with a range of cardiometabolic traits [‘metabolically favourable’, i.e. lower levels of visceral fat and beneficial effects on cardiometabolic factors, for example high-density lipoprotein (HDL) cholesterol and lower triglycerides, and ‘unfavourable’ variants]^29–32^ and lower grey matter volume of the brain,^33^ an understanding of whether and how body fat distribution causes poor cognitive function is warranted.

We aimed to address the above identified knowledge gaps by triangulating findings using two analytical approaches. First, we performed observational analyses investigating the relationship between phenotypic measures of adiposity (BMI, BF%, WHR) and cognitive function [visual memory (VM) and reaction time (RT)] and vice versa. Second, we repeated this analysis within a bidirectional MR framework in which genetic instruments for the adiposity indicators [also including metabolically ‘favourable’/‘unfavourable’ adiposity (FA/UFA)] were used to examine the relationship with VM and RT. This was then repeated in the opposite direction using genetic instruments for RT and VM to examine the relationship with BMI, BF% and WHR.

## Methods

### Study participants

**Figure 1:**
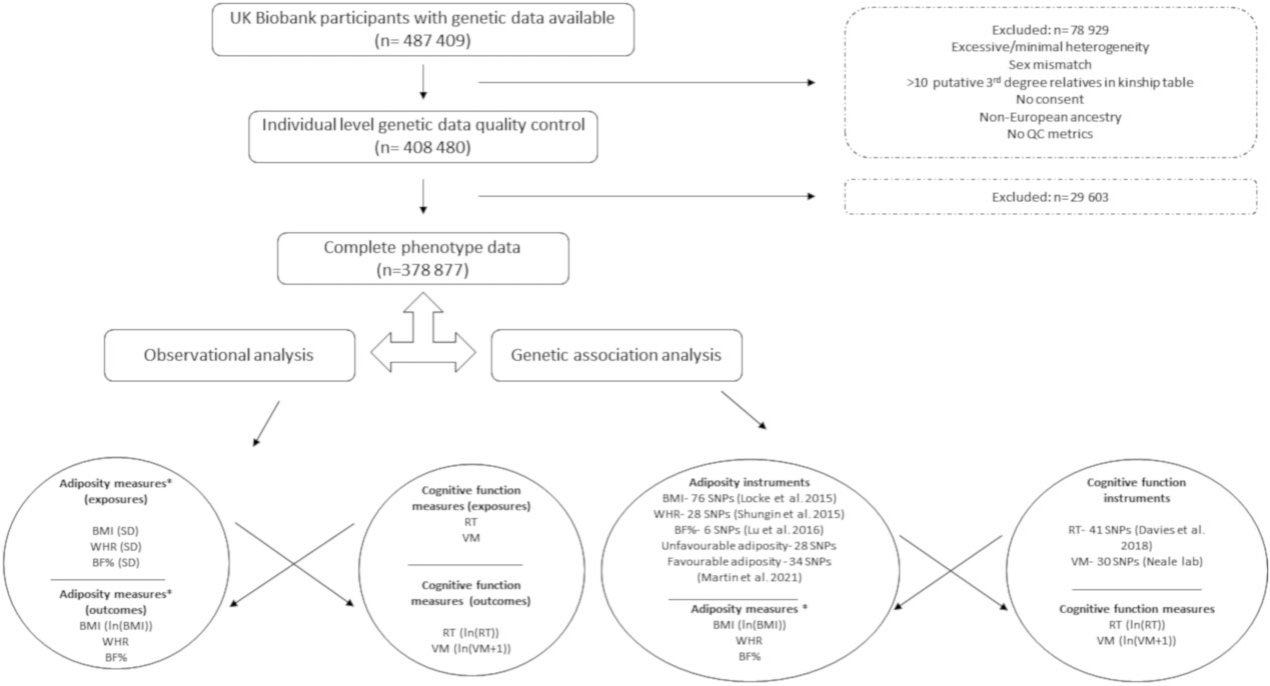
Sample flow diagram and study design illustrating bidirectional approach. QC, quality control; BMI , body mass index; WHR, waist-hip ratio; %BF, body fat percentage; RT, reaction time; VM, visual memory; SNPs, single nucleotide polymorphisms. *Unfavourable/favourable adiposity not ‘measured’ in UKB

UK Biobank (UKB), described in detail elsewhere,^34^ is a large, prospective cohort of individuals aged 40–69 years at recruitment (2006–10) from across the UK.^34^ The sample examined here included 378 877 European ancestry participants with available data on genotypes and all relevant phenotypes (details in Figure 1).

### Study design

We employed a pseudo two-sample bidirectional MR design, using genetic association estimates from individual-level data of UKB participants and genome-wide association study (GWAS) summary statistics (described below), to estimate the causal effect of five indicators of adiposity on two indicators of cognitive function and vice versa.

### Adiposity measures

Adiposity measures were obtained at baseline following standardiezd protocols.^35^ Weight and BF% were measured using a Tanita BC-418 MA body composition analyser; height was measured with a Seca-202 height measure; waist and hip circumferences were measured using a Seca-200 tape measure. BMI (kg/m^2^) and WHR were derived. BMI was positively skewed and so was transformed to the natural logarithmic scale [ln(BMI)] when used as an outcome (details of all parameterizations used are in Supplementary Table S1, available as Supplementary data at *IJE* online).

### Cognitive function measures

At baseline, participants undertook cognitive assessments (described elsewhere^36^). Briefly, for VM, respondents were asked to correctly identify matches from six pairs of cards after they had memorized their positions. The number of incorrect matches (number of attempts made to correctly identify pairs) was recorded. RT (ms) was recorded as mean time participants took to correctly identify matches in a 12-round game of ‘Snap’. A greater number (VM) or time (RT) indicates poorer cognition. Both variables were positively skewed and were transformed using natural logs when considered as outcomes (Supplementary Table S1).

### Confounding variables

Potential confounders were identified from a directed acyclic graph (Supplementary Figures S1 and S2, available as Supplementary data at *IJE* online). They included: the Townsend index^37^ (a measure of area-level deprivation), smoking status, physical activity, age (years), alcohol intake, sleep duration, and comorbidities (type 1 diabetes, stress, depression and chronic fatigue syndrome) (details in Supplementary Methods and Supplementary Table S2, available as Supplementary data at *IJE* online).

### Genetic instrument selection

#### Adiposity

We used 76, 28, 6, 28 and 34 near-independent SNPs for BMI, WHR, BF%, UFA and FA which achieved genome-wide significance (*P *< 5 × 10^−8^) in the respective GWAS.^38–41^ Instrument details are provided in Supplementary Table S3 (available as Supplementary data at *IJE* online). For BMI, WHR and BF%, SNPs were obtained from GWAS which did not include UK Biobank (UKB). For UFA and FA, the GWAS from which SNPs were obtained was performed on UKB participants. Instrument F statistics, obtained from regressions of each phenotype on the respective genetic instrument, ranged from 24.8 (FA) to 91.4 (BMI) and the variance explained ranged from 0.22% (FA) to 1.80% (BMI) (details in Supplementary Table S4, available as Supplementary data at *IJE* online).

#### Cognitive function

For RT, we used 41 SNPs achieving genome-wide significance (*P *< 5 × 10^−8^) in a recent UKB GWAS on 330 069 European-ancestry participants.^42^ For VM, we used 30 SNPs that were downloaded from the Neale laboratory UKB repository^43^ and were obtained from a GWAS performed in 361 194 UKB European-ancestry participants (further instrument details in Supplementary Tables S3 and S4).

Linkage disequilibrium clumping in PLINK1.9 ensured that included SNPs were independent (r^2^ ≤ 0.1, 250 kb, reference haplotype data originated from the publicly released Phase 3 data from the 1000 Genomes Project^44^). Where necessary, beta coefficients were multiplied by −1 to ensure all betas represented an increase in the respective traits; allele harmonization was done to ensure alignment of alleles for SNP-X and SNP-Y associations (details on SNP genotyping, imputation and quality control are in Supplementary Methods, available as Supplementary data at *IJE* online).

### Statistical analyses

#### Observational

We explored observational associations between measured adiposity and cognition and vice versa using linear regression, with and without adjustment for confounders. To ensure comparability across observational and MR analyses, when adiposity measures were used as exposures, we rescaled them so that a 1-unit change represented a 1-standard deviation (SD) change. This was not done when RT and VM were exposures of interest, as their original GWAS were performed on untransformed RT and VM (Supplementary Table S1).

#### Genetic: Bidirectional MR

The following analyses were performed initially with adiposity instruments as exposures and cognitive function measures (RT and VM) as outcomes and then vice versa.

The inverse-variance weighted (MR_IVW_) method was our main MR model. This method estimates the causal effect of the exposure on the outcome by averaging the genetic instruments’ ratio of instrument-outcome (SNP-Y) to instrument-exposure (SNP-X) association estimates using a multiplicative random-effects meta-analysis model.^45^ We quantify the extent of heterogeneity between SNP-specific causal estimates by reporting the I^2^ statistic. SNP-Y associations were estimated using linear regressions, adjusted for 10 genetic principal components. SNP-X associations were extracted from the original GWAS.^38–43^ We performed two MR sensitivity analyses: Mendelian randomization-Egger (MR_Egger_)^46^ and weighted median estimator (MR_WME_).^47^ MR_Egger_ yields an intercept term which indicates the presence of unbalanced horizontal pleiotropy (i.e. if genetic instruments are associated with the outcome via pathways other than via the exposure); MR_WME_ provides more robust estimates when up to 50% of the genetic variants are invalid. We report $I_{\text{GX}}^{2}$ which quantifies the magnitude of regression dilution bias in the context of MR_Egger_^48^ (further details on MR methods are in Supplementary Methods). To account for the high number of comparisons being made between adiposity and cognition (and vice versa) (*n* = 16 tests), we applied a Bonferroni adjustment to all *P*-value thresholds (i.e. *P-*value threshold/number of tests (16); *P* <0.05 corresponds to *P *<0.003125, and *P* <0.01 corresponds to *P* <0.000625).

#### Sensitivity analyses

For results from MR analyses to be valid, three key assumptions must be met: (i) genetic variants should be robustly associated with the exposure; (ii) genetic variants should be independent of confounding factors of the relationship in question; (iii) the association between genetic variants for the exposure and the outcome must only operate via the exposure under study. Here we provide brief details regarding how these assumptions were assessed (further details in Supplementary Methods). We explored the validity of our instruments by testing associations between SNPs and above-described potential confounders, applying a Benjamini-Hochberg false-discovery rate of 0.05 to account for multiple testing. Where associations were observed, MR analyses were re-run excluding potentially invalid SNPs. In addition, when the MR_Egger_ intercept indicated pleiotropy (*P * < 0.05), we undertook further analyses. Outlying SNPs and those with a large influence on the estimates were identified by (i) funnel plots and (ii) Cook’s Distance.^49^ We then reran our analyses removing the identified SNPs.

As the SNPs used to derive the UFA, FA, RT and VM instruments were constructed using GWAS including UKB, we calculated the extent to which genetic effect sizes were biased as a result of ‘winner’s curse’ (i.e. overestimation of causal effects in a one-sample setting),^50^ using established methods.^51^ For the RT and VM instruments, we further investigated the extent of this bias by employing a split-sample strategy, as has been done elsewhere.^52^ We split the data randomly into two samples: A and B, with N_A_ = 189 439 and N_B_ = 189 438. We calculated individual SNPs’ genetic association with the exposure (SNP-X) and the outcome (SNP-Y) by running simple linear or logistic regressions in each sample. For MR analyses, we used SNP-X from sample A and SNP-Y from sample B (A on B) and vice versa (B on A). Finally, we meta-analysed the two MR estimates (Meta A & B) and compared these with the MR estimates from our main analysis. It was not possible to employ the split-sample strategy for analyses involving UFA and FA (as either exposures or outcomes), as these phenotypes were not observable in UKB, to derive estimates of either SNP-X_(favourable or unfavourable)_ or SNP-Y_(favourable or unfavourable)_ betas.

We used Stata16 and PLINK1.9 and 2.0 for data processing and statistical analyses. MR analyses were performed using *mrrobust* in Stata.^53^

## Results

Participants’ mean age was 56.7 (SD = 8) years (Table 1). Males had a higher BMI and WHR and lower BF% compared with females. Median RT was 535 ms (25th, 75th centile: 477, 606) and median number of incorrect matches (i.e. VM) was 3 (25th, 75th centile: 2,5).

**Table 1:** Sample characteristics (*n* = 378 877)

Variable	*N*(%)/median (25th, 75th centile)
**Sociodemographic characteristics**	
Sex	
Male	174 968 (46.2)
Female	203 909 (53.8)
Age at recruitment (years)^a^	56.7 (8.0)
Townsend deprivation index^b^	−2.4 (−3.8, −0.0)
Currently smoking	
No	341 833 (90.2)
Yes	37 044 (9.8)
Alcohol consumption	
Less than daily	297 061 (78.4)
Almost/daily	81 816 (21.6)
Physical activity	
Active (vigorous activity ≥4x/wk)	70 012 (18.5)
Inactive (vigorous activity <4x/wk)	308 865 (81.5)
Sleep duration per night (h)	7.1 (1.1)
Comorbidities present^c^	
No	355 781 (93.9)
Yes	23 096 (6.1)
**Adiposity indicators**	
BMI (kg/m^2^)	
Male	27.3 (25.0, 30.0)
Female	26.0 (23.4, 29.5)
BF%^c^	
Male	25.2 (5.8)
Female	36.5 (6.8)
Waist-hip ratio^c^	
Male	0.9 (0.1)
Female	0.8 (0.1)
**Cognitive function**	
Visual memory (number of incorrect matches)	3 (2, 5)
Reaction time (ms)	535 (477, 606)

BMI, body mass index; %BF, body fat percentage; SD, standard deviation; wk, weeks.

a Summarized as mean(SD).

b A higher index indicates more deprivation;.

c Type 1 diabetes, stress, depression and chronic fatigue syndrome (see Supplementary Methods and Supplementary Table S2, available as Supplementary data at *IJE* online for details).

### Adiposity to cognitive function

#### Observational analysis

In adjusted models, a 1-SD higher BF% was associated with higher, i.e. slower, RT and with a lower number of incorrect matches, i.e. better VM (Table 2; Supplementary Figures S3 and S4, available as Supplementary data at *IJE* online). Higher BMI and WHR, were associated with faster RT and better VM, e.g. a 1-SD higher BMI was associated with 0.23% faster RT [β = −0.23%; 95% confidence interval (CI): −0.29%, −0.18%] and 1.83% lower VM score (β = −1.83%; 95% CI: −2.03%, −1.63%).

**Table 2:** Estimated causal effects of adiposity^a^ on cognitive function (*n *= 378 877)

**A. Percentage difference (95% CI, *P*-value** ^c^ **) in reaction time by adiposity indicators**										
	BF%		BMI		WHR		UFA		FA	
**Observational analyses**										
Unadjusted	2.02 (1.96, 2.08)	<0.001	0.22 (0.16, 0.28)	<0.001	0.20 (0.14, 0.26)	<0.001	–		–	
Adjusted^b^	1.24 (1.19, 1.30)	<0.001	−0.23 (−0.29,-0.18)	<0.001	−0.79 (−0.85, −0.74)	<0.001	–		–	
**MR analyses**										
Number SNPs	6		76		28		28		34	
IVW	−1.28 (−2.29, −0.25)	*0.02*	−0.63 (−1.33, 0.07)	0.08	−0.06 (−0.82, 0.70)	0.08	−0.96 (−2.30, 0.39)	0.16	−0.20 (−1.94, 1.58)	0.83
I^2^	0.14		0.70		0.20		0.62		0.58	
WME	−0.93 (−2.09, 0.26)	0.13	−0.29 (−0.98, 0.41)	0.42	0.08 (−0.87, 1.04)	0.87	−0.69 (−2.04, 0.68)	0.32	−1.39 (−3.29, 0.54)	0.16
MR-Egger	0.60 (−3.58, 4.95)	0.78	0.54 (−1.18, 2.30)	0.54	−0.27 (−3.66, 3.23)	0.88	1.82 (−2.67, 6.52)	0.43	−0.14 (−4.87, 4.83)	0.96
*P-*pleiotropy	0.37		0.15		0.90		0.21		0.98	
$I_{\text{GX}}^{2}$	0.69		0.89		0.64		0.91		0.88	

**B. Percentage difference (95% CI, *P*-value** ^c^ **) in visual memory by adiposity indicators**										
	BF%		BMI		WHR		UFA		FA	
**Observational analyses**										
Unadjusted	0.59 (0.38, 0.79)	<0.001	−1.29 (−1.49, −1.09)	<0.001	0.51 (0.31, 0.71)	<0.001	–		–	
Adjusted^b^	−0.43 (−0.63, −0.22)	<0.001	−1.83 (−2.03, −1.62)	<0.001	−0.98 (−1.18, −0.77)	<0.001	–		–	
**MR analyses**										
Number SNPs	6		76		28		28		34	
IVW	−2.53 (−9.00, 4.39)	0.46	−0.41 (−2.54, 1.76)	0.71	−1.09 (−4.14, 2.05)	0.49	−3.55 (−7.30, 0.35)	0.07	0.62 (−4.94, 6.51)	0.83
I^2^	0.77		0.63		0.44		0.47		0.51	
WME	−2.79 (−7.34, 1.99)	0.25	−1.05 (−3.30, 1.25)	0.37	−2.67 (−6.08, 1.01)	0.16	−3.14 (−7.51, 1.43)	0.18	−0.54 (−7.30, 6.72)	0.88
MR-Egger	−16.64 (−36.09, 8.73)	0.18	0.24 (−5.00, 5.77)	0.93	−15.92 (−25.90, −4.60)	0.01	5.75 (−7.16, 20.45)	0.40	−1.22 (−15.51, 15.48)	0.88
*P*-pleiotropy	0.23		0.80		0.01		0.15		0.80	
$I_{\text{GX}}^{2}$	0.69		0.89		0.64		0.91		0.88	

IVW, inverse-variance-weighted; WME, weighted median estimator; MR-Egger, Mendelian randomization Egger regression; %BF, body fat percentage; BMI, body mass index; WHR, waist-hip ratio; UFA, unfavourable adiposity; FA, favourable adiposity; MR, Mendelian randomization; SNPs, single nucleotide polymorphisms.

a Associations between measured and genetically predicted increases in one standard deviation in adiposity and percent difference in reaction time (ms) and visual memory (number incorrect matches).

b Adjusted for deprivation, age at recruitment, smoking status, alcohol consumption, physical activity, sleep duration and comorbidities.

c *P*-values need to be considered after correcting for multiple testing using a Bonferroni adjustment (number of tests = 16; i.e. *P *<0.05 corresponds to *P *<0.003125 and *P *<0.01 corresponds to *P *<0.000625).

#### MR analysis

In contrast to observational findings, two of the three MR analyses (MR_IVW_ and MR_WME_) found higher BF% was associated with faster RT (Table 2; Supplementary Figures S3 and S4, available as Supplementary data at *IJE* online). For all other adiposity-cognitive function associations, at least two of the three MR analyses agreed with adjusted observational findings, although in most situations confidence intervals were wide and included the null. For example, a 1-SD higher BMI was associated with 0.63% faster RT (β = −0.63%; 95% CI: −1.33%, 0.07%) (MR_IVW_ analysis). Estimates from at least two of the MR analyses using ‘unfavourable’ and ‘favourable’ adiposity instruments indicated higher adiposity was associated with faster RT and lower VM score, again with wide confidence intervals which included the null.

### Cognitive function to adiposity

#### Observational analysis

In adjusted models, higher (i.e. worse) RT was associated with higher BF% and lower WHR and BMI; higher (worse) VM was associated with lower BF%, WHR and BMI (Table 3; Supplementary Figures S5–S7, available as Supplementary data at *IJE* online). For example, a 1-ms higher RT was associated with a 0.003% lower BMI (β = −0.003%; 95% CI: −0.003%, −0.002%).

**Table 3:** Estimated causal effects of cognitive function^a^ on adiposity (*n* = 378 877)

A. **Difference (95% CI, *P*-value**^c^**) in BF% by cognitive function**				
	RT		VM	
**Observational analyses**				
Unadjusted	0.01 (0.01, 0.01)	<0.001	−0.01 (−0.01, 0.003)	0.24
Adjusted^b^	0.005 (0.004, 0.005)	<0.001	−0.05 (−0.06, −0.04)	<0.001
**MR analyses**				
Number of SNPs	41		30	
IVW	−0.29 (−1.77, 1.20)	0.71	−1.32 (−1.88, −0.77)	<0.001
I^2^	0.81		0.75	
WME	−0.76 (−1.91, 0.39)	0.17	−1.45 (−1.93, −0.96)	<0.001
MR-Egger	−8.51 (−24.18, 7.16)	0.29	−2.49 (−6.21, 1.23)	0.19
*P-*pleiotropy	0.30		0.54	
$I_{\text{GX}}^{2}$	0.61		0.00	


B. **Difference (95% CI, *P-*value**^c^**) in WHR by cognitive function**				
	RT		VM	
**Observational analyses**				
Unadjusted	1*10^−5^ (8*10^−6^, 1*10^−5^)	<0.001	4*10^−4^ (3*10^−4^, 5*10^−4^)	<0.001
Adjusted^b^	−3*10^−5^ (−3*10^−5^, −3*10^−5^)	<0.001	−2*10^−4^ (−3*10^−4^, −2*10^−4^)	<0.001
**MR analyses**				
Number of SNPs	41		30	
IVW	−0.0004 (−0.01, 0.01)	0.95	−0.005 (−0.01, 0.002)	0.15
I^2^	0.68		0.80	
WME	0.001 (−0.01, 0.01)	0.82	−0.002 (−0.01, 0.003)	0.43
MR-Egger	0.001 (−0.13, 0.13)	0.99	−0.01 (−0.05, 0.03)	0.67
*P*-pleiotropy	0.99		0.83	
$I_{\text{GX}}^{2}$	0.61		0.00	

**C. Percent difference (95% CI, *P*-value^c^) in BMI by cognitive function**				
	RT		VM	
**Observational analyses**				
Unadjusted	0.001 (0.001, 0.002)	<0.001	−0.11 (−0.13, −0.10)	<0.001
Adjusted^b^	−0.003 (−0.003, −0.002)	<0.001	−0.16 (−0.18, −0.15,	<0.001
**MR analyses**				
Number of SNPs	41		30	
IVW	−0.39 (−4.07, 3.43)	<0.84	−3.57 (−5.15, −1.84)	<0.001
I^2^	0.89		0.90	
WME	−0.86 (−3.26, 1.60)	0.49	−2.71 (−3.70, −1.63)	<0.001
MR-Egger	−7.58 (−38.19, 38.20)	0.70	−11.01 (−20.42, −0.48)	0.04
*P*-pleiotropy	0.71		0.15	
$I_{\text{GX}}^{2}$	0.61		0.00	

IVW, inverse-variance weighted; WME, weighted median estimator; MR-Egger, Mendelian randomization Egger regression; %BF, body fat percentage; BMI, body mass index; WHR, waist-hip ratio; RT, reaction time; VM, visual memory; MR, Mendelian randomization; SNPs, single nucleotide polymorphisms.

a Associations between measured and genetically predicted increases in reaction time (ms) and visual memory (number incorrect matches) on adiposity.

b Adjusted for deprivation, age at recruitment, smoking status, alcohol consumption, physical activity, sleep duration and comorbidities.

c *P*-values need to be considered after correcting for multiple testing using a Bonferroni adjustment (number of tests = 16; i.e. *P *<0.05 corresponds to *P *<0.003125 and *P *<0.01 corresponds to *P *<0.000625).

#### MR analyses

MR estimates of the RT-adiposity associations generally indicated that a higher (i.e. worse) RT was associated with lower BF% and BMI, but estimates had wide confidence intervals which included the null. All three MR analyses were directionally consistent with the observational analysis for the association between RT and BMI (Table 3; Supplementary Figures S5–S7). For example, a 1-ms higher RT was associated with a 0.86% (β = −0.86%; 95% CI: −3.26, 1.60) lower BMI (MR_WME_ analysis). For the RT-BF% and RT-WHR associations, although MR associations generally agreed with each other, they were directionally inconsistent with adjusted observational findings. All three MR analyses for the effect of VM on BF%, WHR and BMI were directionally consistent with each other and with the adjusted observational analyses, indicating that higher (worse) VM resulted in lower adiposity. For example, a 1-unit worse VM score was associated with a 1.32% (β = −1.32%; 95% CI: −1.88, −0.77) and 3.57% (β = −3.57%; 95% CI: −5.15, −1.84) lower absolute BF% and relative BMI, respectively (MR_IVW_ analyses). Whereas a higher (worse) VM score also resulted in a lower WHR in all three MR analyses, confidence intervals included the null.

#### Sensitivity analyses

When removing SNPs associated with confounders from instruments, associations from adiposity to cognition (in particular for VM) changed direction (Supplementary Table S5, available as Supplementary data at *IJE* online). In the other direction, whereas some associations from cognition to adiposity (e.g. VM to BF% and BMI) were consistent with the main MR analysis, others (e.g. RT to BF%) were not (Supplementary Table S6, available as Supplementary data at *IJE* online). In addition, as per the main analyses, many of the confidence intervals were wide and included the null.

There was one instance of horizontal pleiotropy: for the effect of WHR on VM (MR_Egger_*P*-value_intercept_ = 0.01). This pleiotropic effect remained after removing 11 SNPs which were associated with confounding variables (MR_Egger_*P*-value_intercept_ = 0.003) (Supplementary Table S5). Funnel plots and the calculation of Cook’s Distance identified four potentially pleiotropic SNPs (rs1121980, rs12549058, rs2075650 and rs9491696). When the analysis was rerun without these SNPs, there was no evidence of pleiotropy (MR_Egger_*P*-value_intercept_ = 0.42), but associations were directionally inconsistent with those reported in the main analysis (both observational and MR estimates), though confidence intervals remained wide and included the null (Supplementary Table S7, available as Supplementary data at *IJE* online).

Estimated biases due to sample overlap were small: absolute bias <0.005; type-1 error rate = 0.05 for all outcomes (Supplementary Table S8, available as Supplementary data at *IJE* online). Results from the split-sample strategy in which RT and VM were the exposures are presented in Supplementary Table S9 (available as Supplementary data at *IJE* online). The meta-analysis of estimates from MR (A on B) and MR (B on A) were smaller, but in line with those reported above.

## Discussion

We investigated evidence for causal links between adiposity and cognitive function in UK Biobank using several complementary approaches, and found important differences in terms of the postulated direction of association. Using a bidirectional MR design, we show the effect of adiposity on cognitive function is likely not to be causal. In the other direction, we found little evidence to support causal links between RT and adiposity; however, our findings do strengthen the evidence base for causal links between poor VM and lower adiposity.

In the direction adiposity to cognition, observational estimates for the effect of adiposity on RT were either attenuated (BF%) or flipped direction (BMI, WHR) upon adjustment for confounders, which likely reflects the impact of confounding in the unadjusted estimates. MR estimates were imprecisely estimated and, in almost all instances, included the null. Furthermore, estimates changed direction in the main compared with the sensitivity analyses. The lack of effect of adiposity on cognition agrees with the null MR findings between BMI and verbal-numerical reasoning observed by Hagenaars and colleagues^26^ and also a recent bidirectional MR study by Wang *et al.*^27^ who observed no effect of BMI on verbal-numerical reasoning in both European and Asian populations. The authors did conclude that an inverse relationship between WHR_adj_BMI and cognitive performance was evident, though covariable-adjusted summary associations such as WHR_adj_BMI should be interpreted with caution as such instruments have been found to introduce bias into MR analyses.^28^ Here we consolidate and extend previous work, providing evidence of a null effect, on two different measures of cognitive function (reaction time and visual memory), of total (BMI, BF%) and central (WHR) adiposity, as well as adiposity associated with favourable and unfavourable metabolic profiles. The consistency of findings across MR studies using different adiposity and cognitive function measures supports a likely null effect of adiposity on cognitive function.

In the direction cognition to adiposity, MR estimates were generally consistent in their direction as to the effects of RT and VM on adiposity traits, such that worse RT and VM resulted in lower BF% and BMI. For RT, however, all confidence intervals included the null. Our findings are in contrast with previous observational findings suggesting an association between worse cognitive function and subsequent higher BMI,^20^^,^^22^^,^^23^ and this may be related to the different periods of observation across the studies (e.g. childhood vs adulthood). Estimates from MR studies exploring the effect of cognitive ability on adiposity are conflicting. Whereas Wang *et al*.^27^ reported a that higher cognitive function caused lower BMI, the study by Davies *et al*.^54^ concluded that there was no direct effect of intelligence on BMI. That we observed some evidence suggesting that poorer visual memory (i.e. lower cognitive function) caused lower BMI and BF%, could reflect the different indicators of cognitive ability and thus the cognitive phenotype being examined (e.g. both Wang *et al*. and Davies *et al*. used verbal-numerical reasoning).

Our findings do, however, concur with a recent longitudinal study that observed that those with lower cognitive function at age 50 years demonstrated greater reductions in BMI over the subsequent 40 years.^55^ Furthermore, as poor VM and/or RT are precursors of Alzheimer’s disease (AD),^56^ our findings extend a previous bidirectional MR study which observed no effect of a genetic predisposition to higher BMI on risk of AD but found that those with increased risk of AD had lower BMI.^57^ Poorer VM, in particular, may represent an early expression of AD decades prior to diagnosis,^56^ and thus our findings of poorer VM resulting in a lower adiposity (including BMI) suggests that the effect of cognitive abnormalities on reduced adiposity may manifest at much less severe levels of cognitive impairment.

The major strength of our study is that by using a bidirectional design, we have been able to establish the direction of causal effects between adiposity and cognitive function. By performing both observational and MR analyses and via the use of multiple indicators of adiposity and cognitive function, we have also been able to triangulate findings to more comprehensively explore the adiposity-cognitive function relationship. Within our bidirectional MR framework, we used three different methods which have distinct strengths and assumptions.^46^^,^^47^ The general agreement across these different analytical approaches, particularly for the VM-adiposity relationship, strengthens the causal interpretation of the findings.

We acknowledge some limitations. Our observational analysis was cross-sectional; direction of causality cannot be inferred from such study designs. The genetic variants included in our UFA, FA, RT and VM instruments were obtained from GWAS that contained UKB participants, potentially leading to an overestimation of genetic associations (‘winner’s curse’^51^). We investigated the extent of the bias resulting from sample overlap^51^ and found it to be small. Furthermore, when we investigated the extent of this bias (for the RT and VM instruments) by employing a split-sample strategy, we observed estimates which were directionally consistent with those from the full sample. Relatedly, in attempting to mitigate the risk of sample overlap, for our BMI, BF% and WHR instruments we only included genetic variants obtained from GWAS excluding UKB participants. This resulted in a smaller number of SNPs in our instruments than otherwise would have been possible, which likely reduced the power of our MR analyses and may have contributed to some weak instrument bias.^58^ Another consideration is measurement error in our phenotypic data. The VM assessment performed less well in terms of reliability, compared with the other cognitive function assessments in UKB.^36^ Additionally, VM was subject to a ‘floor’ effect.^59^ Finally, there is evidence of selection bias into UKB,^60^ which has to the potential to induce collider bias and produce biased estimates.^61^

Our results have important public health implications. In light of our finding of a potential causal effect of poorer cognitive function over a lifetime on lower adiposity levels in mid-to-late adulthood, practitioners should be vigilant to unexplained reductions in adiposity in mid/later adulthood as these may represent a symptom of reductions in cognitive function.

## Conclusions

We demonstrate that the effect of adiposity on cognitive function is likely not to be causal. In the reverse direction, whereas we had little evidence to support causal effects of RT on adiposity, we observed a consistent effect of VM on adiposity, such that worse VM resulted in lower BF%, WHR and BMI. Findings should be interpreted in the context of the limitations of the study and should be triangulated using other cognitive outcomes and complementary methods to determine their robustness.

## Ethics approval

Participants provided informed consent; ethical approval was given by the North-West Multicentre Research Ethics Committee.

## Supplementary Material

dyad043_Supplementary_Data

## Data Availability

The UK Biobank data are publicly available to all bona fide researchers at [https://www.ukbiobank.ac.uk].
