# Correction to: The relationship between adiposity and cognitive function: a bidirectional Mendelian randomization study in UK Biobank

**DOI:** 10.1093/ije/dyad070

**Published:** 2023-05-18

This is a correction to: Tom Norris, Antoine Salzmann, Albert Henry, Victoria Garfield, Snehal M Pinto Pereira, The relationship between adiposity and cognitive function: a bidirectional Mendelian randomization study in UK Biobank, *International Journal of Epidemiology*, 2023, dyad043, https://doi.org/10.1093/ije/dyad043

In the originally published version of this manuscript the “Author Notes” in the online version mistakenly said “Albert Henry and Snehal M Pinto Pereira Joint senior authors.” This should have stated “Victoria Garfield and Snehal M Pinto Pereira Joint senior authors.”

This error has been corrected.

