# Supplementary material for: The relationship between adiposity and cognitive function: a bidirectional Mendelian randomization study in UK Biobank
Source: Int J Epidemiol. 2023 Apr 8;52(4):1074–85. doi: 10.1093/ije/dyad043 (PMC10396406; doi:10.1093/ije/dyad043)
Supplement: dyad043_Supplementary_Data [file dyad043_supplementary_data.docx]

**Supplementary Material: The relationship between adiposity and cognitive function: A Mendelian randomization study in UK Biobank**

**Contents**

[**Participant selection** 3](#_Toc129338978)

[**Variable ascertainment** 3](#_Toc129338979)

[**Covariates** 3](#_Toc129338980)

[**Genotyping, Imputation, and quality control** 3](#_Toc129338981)

[**Mendelian randomization (MR) analysis methods and assumptions** 4](#_Toc129338982)

[**Inverse-variance weighted** 5](#_Toc129338983)

[**MR-Egger regression** 5](#_Toc129338984)

[**Weighted median estimator (WME)** 5](#_Toc129338985)

[**Supplementary tables** 7](#_Toc129338986)

[**Supplementary table S1: Parameterisation of exposures/SNP-X* and outcomes** 7](#_Toc129338987)

[**Supplementary table S2: List of UK Biobank codes for comorbidities** 8](#_Toc129338988)

[**Supplementary Table S3: Summary statistics describing SNP-X associations for the 5 adiposity and 2 cognitive function instruments** 9](#_Toc129338989)

[**Supplementary Table S4: Details of genetic instruments used** 14](#_Toc129338990)

[**Supplementary table S5: Removing adiposity SNPs associated with potential confounders** 15](#_Toc129338991)

[**Supplementary table S6: Removing cognitive function SNPs associated with potential confounders** 16](#_Toc129338992)

[**Supplementary Table S7: Removing potentially pleiotropic SNPs from analysis of associations between WHR and VM** 17](#_Toc129338993)

[**Supplementary Table S8: Estimation of bias due to sample overlap** 18](#_Toc129338994)

[**Supplementary Table S9. Causal effects of cognitive function on adiposity: split sample strategy*** 19](#_Toc129338995)

[**Supplementary Figures** 20](#_Toc129338996)

[**Supplementary Figure S1: Adiposity-cognitive function directed acyclic graph (DAG)*** 20](#_Toc129338997)

[**Supplementary Figure S2: Cognitive function-adiposity directed acyclic graph (DAG)*** 21](#_Toc129338998)

[**Supplementary Figure S3: Observational and MR estimates of association between adiposity and reaction time** 22](#_Toc129338999)

[**Supplementary Figure S4: Observational and MR estimates of association between adiposity and visual memory** 23](#_Toc129339000)

[**Supplementary Figure S5: Observational and MR estimates of association between cognitive function and body fat percentage** 24](#_Toc129339001)

[**Supplementary Figure S6: Observational and MR estimates of association between cognitive function and waist-hip ratio** 25](#_Toc129339002)

[**Supplementary Figure S7: Observational and MR estimates of association between cognitive function and body mass index** 26](#_Toc129339003)

[**References** 27](#_Toc129339004)

# **Participant selection**

At the time of our study, UK Biobank had genetic data available for 487,409 participants. We applied individual-level quality control (QC) to exclude participants with excessive / minimal heterozygosity, sex mismatch, excessive genetic relatedness (more than 10 putative third-degree relatives in the kinship table), no consent, non-European ancestry, and missing QC metrics, leaving 408,480 participants for analysis. We further excluded participants with no phenotype data (N = 29,603), leaving 378,877 participants for subsequent analyses (**Figure 1**).

# **Variable ascertainment**

## **Covariates**

Potential confounders were identified from a directed acyclic graph (DAG) constructed using the online tool DAGitty (<https://www.dagitty.net>). Information on participants’ age, sex, smoking status, alcohol intake frequency and physical activity level were collected using a self-reported questionnaire at baseline. Townsend deprivation index was assigned based on postcode as a continuous measure, where a higher index indicates more deprivation. Smoking status was a dichotomous variable defined as not currently smoking vs. currently smoking. Alcohol intake frequency was based on the question *‘how often do you drink’*, from which a dichotomous variable was derived representing: alcohol intake less than daily vs. alcohol intake daily/almost daily. Physical activity was based on responses to the question *‘Number of days per week of vigorous physical activity lasting at least 10 minutes’*, from which a dichotomous variable was derived: active (at least 4 days/week vigorous physical activity lasting at least 10 minutes) vs. inactive (less than 4 days/week vigorous physical activity lasting at least 10 minutes). Sleep duration (hours) was self-reported by study participants based on the standardized question: “About how many hours sleep do you get in every 24 h? (Please include naps)”. Comorbidities were identified as a binary variable from baseline self-reported illness data using UK Biobank code list (**Supplementary Table 2**).

# **Genotyping, Imputation, and quality control**

Genotyping in UK Biobank was performed using two customised genome-wide arrays, with a genome-wide imputation performed using combined reference panels from UK10K, 1000 Genomes phase 3, and Haplotype Reference Consortium (HRC), resulting in 93,095,623 autosomal variants (1). We further applied quality control to exclude variants with the following criteria: Fisher information <0.3, missing call rate ≥ 5%, or MAF outside of 0.01–1 range.

# **Mendelian randomization (MR) analysis methods and assumptions**

MR analysis uses genetic variants as proxies for exposure of interest to estimate the causal association between the exposure and outcome of interest in an instrumental variable (IV) framework. A conventional MR makes assumptions that genetic instruments for the exposure must be:

- Robustly associated with the exposure. This assumption is reasonable as the genetic variants for each of the traits used in our study achieved genome-wide significance in their respective (large-scale) genome wide association study (GWAS).
- Associated with the outcome only through exposure. This assumption was assessed using MR-Egger.
- Independent of confounders that influence the exposure and outcome after conditioning on observed confounders (2). We checked this assumption by regressing adiposity and cognitive function single nucleotide polymorphisms (SNPs) on multiple confounders (area-level deprivation, smoking status, physical activity, age at recruitment, alcohol intake and comorbidities) using linear or logistic regression (as appropriate). We applied a Benjamini-Hochberg false discovery rate (BH-FDR) of 0.05 to account for multiple testing.

In this study, we utilised a bidirectional multi-instrument, two-sample MR approach (where summary statistics for genetic associations with the exposure and the outcome, typically estimated in two independent samples, were used to calculate the MR estimate). Specifically, we used the following methods:

## **Inverse-variance weighted**

For uncorrelated genetic variant $j$, the causal estimate $\hat{\beta}_{IVW}$ can be calculated by averaging the ratio between variant-outcome association estimate $\hat{\beta}_{Y_{j}}$ and variant-exposure association estimate $\hat{\beta}_{X_{j}}$ using the inverse-variance weighted formula for multiplicative random-effect meta-analysis model, which yields:

$$\hat{\beta}_{IVW}= \frac{\sum_{j} \hat{\beta}_{Y_{j}}\hat{\beta}_{X_{j}}\sigma_{Y_{j}}^{-2}}{\sum_{j} \hat{\beta}_{X_{j}}^{2}\sigma_{Y_{j}}^{-2}}$$

where $\hat{\beta}_{Y_{j}}$ (or $\hat{\beta}_{X_{j}}$) and $\sigma_{Y_{j}}$ are the coefficient and standard error from regression of, e.g., genetic variant $j$ on cognitive outcomes $Y$ and genetic variant $j$ on adiposity $X$. (3)

## **MR-Egger regression**

The MR-Egger regression performs a weighted linear regression of $\hat{\beta}_{Y_{j}}$ on the $\hat{\beta}_{X_{j}}$, using the $\sigma_{Y_{j}}^{-2}$ as weights and with unconstrained intercept^11^. The causal estimate $\hat{\beta}_{E}$ is obtained from the model:

$$\hat{\beta}_{Y_{j}}= \hat{\alpha}_{E}+\hat{\beta}_{E} \hat{\beta}_{X_{j}}$$

The intercept term $\hat{\alpha}_{E}$ denotes the estimated horizontal pleiotropic effect across genetic variants, and thus, the associated *P*-value is indicative of overall horizontal pleiotropy. The MR-Egger estimates typically have low power, but we used this method to detect horizontal pleiotropy (4).

Both the IVW and MR-Egger models make several additional assumptions, including NO Measurement Error (NOME), InSIDE (Instrument Strength Independent of Direct Effect), and VIS (Variation in Instrument Strength), as detailed elsewhere (5).

## **Weighted median estimator (WME)**

In the weighted median estimator, first the causal estimate $\hat{\beta}$ of each variant $j$ is calculated with the ratio method as $\hat{\beta}_{j}={\hat{\beta}_{Y_{j}}}/{\hat{\beta}_{X_{j}}}$. Then, $\hat{\beta}_{j}$ are sorted (so that $\hat{\beta}_{1}< \hat{\beta}_{2}<\ldots< \hat{\beta}_{J}$) and standardised weight $w_{j}$ is assigned to the $j$th-ordered ratio estimate. The weights are calculated using the inverse variance of the ratio estimates as $\acute{w}_{j}=\hat{\beta}_{X_{j}}\sigma_{Y_{j}}^{-2}$. The standardised weights are $w_{j}={\acute{w}_{j}}/{\sum_{j} \acute{w}_{j}}$ and their sum is $s_{j}= 1$.

If $k$ denotes the largest integer such that the sum of weights up to and including the $k$th estimate ($s_{k}= \sum_{j\leq k} w_{j}$ ) is <0.5, the causal estimate from weighted median method $\hat{\beta}_{WME}$ can be calculated by interpolation between the $k$th and $(k+1)$th ratio estimates as follows:

$$\hat{\beta}_{WME}= \hat{\beta}_{k}+\left( \hat{\beta}_{k+1}- \hat{\beta}_{k} \right) \times\frac{0.5- s_{k}}{s_{k+1}- s_{k}}$$

This approach should provide a consistent estimate given that at least 50% of the weights are derived from valid variants and is more robust to violation of the untestable InSIDE assumption (6, 7).

# **Supplementary tables**

##

## **Supplementary table S1: Parameterisation of exposures/SNP-X* and outcomes**

| Exposure-Outcome association | Exposure/SNP-X parameterisation* | Outcome parameterisation |
| --- | --- | --- |
| *Adiposity to cognition* |  |  |
| BF%- RT | Standard deviation | ln(RT) |
| WHR- RT | Standard deviation | ln(RT) |
| BMI- RT | Standard deviation | ln(RT) |
| BF%- VM | Standard deviation | ln(VM+1) |
| WHR- VM | Standard deviation | ln(VM+1) |
| BMI- VM | Standard deviation | ln(VM+1) |
| *Cognition to adiposity* |  |  |
| RT- BF% | Untransformed | Untransformed |
| RT- WHR | Untransformed | Untransformed |
| RT- BMI | Untransformed | ln(BMI) |
| VM- BF% | Untransformed | Untransformed |
| VM- WHR | Untransformed | Untransformed |
| VM- BMI | Untransformed | ln(BMI) |

*’Exposures’: when referring to observational analysis; ‘SNP-X’: when referring to MR analyses and refers to the scaling of the trait (i.e., X) on which the original GWAS was performed; BF%=Body fat percentage; WHR=Waist-hip ratio; BMI=Body mass index; RT=Reaction time; VM=Visual memory; ln=natural logarithm; SNP-single nucleotide polymorphism

## **Supplementary table S2: List of UK Biobank codes for comorbidities**

| **Categories** | **Code*** | **Illneses** |
| --- | --- | --- |
| **Diabetes & Metabolic** | 1222 | Type 1 diabetes |
| **Psychiatry** | 1614 | Stress |
|  | 1286 | Depression |
|  | 1482 | Chronic fatigue syndrome |

^*^UK Biobank data-coding 6 for non-cancer illnesses: <http://biobank.ctsu.ox.ac.uk/crystal/coding.cgi?id=6>

## **Supplementary Table S3: Summary statistics describing SNP-X associations for the 5 adiposity and 2 cognitive function instruments**

##

| **Instrument** | **SNP ID** | **Chromosome** | **Effect allele** | **Other allele** | **Beta*** | **SE** | ***P*-value** |
| --- | --- | --- | --- | --- | --- | --- | --- |
| BF% | rs1558902 | 16 | A | T | 0.051 | 0.005 | 1.10E-25 |
| BF% | rs2943652 | 2 | C | T | 0.030 | 0.005 | 1.30E-09 |
| BF% | rs6738627 | 2 | A | G | 0.030 | 0.005 | 1.80E-08 |
| BF% | rs693839 | 13 | C | T | 0.030 | 0.005 | 9.30E-09 |
| BF% | rs6857 | 19 | C | T | 0.053 | 0.009 | 6.80E-10 |
| BF% | rs9906944 | 17 | C | T | 0.035 | 0.006 | 1.90E-08 |
| WHR | rs2765539 | 1 | T | C | 0.027 | 0.004 | 1.08E-12 |
| WHR | rs1011731 | 1 | A | G | -0.019 | 0.003 | 1.07E-08 |
| WHR | rs4846565 | 1 | A | G | -0.023 | 0.004 | 4.75E-11 |
| WHR | rs10195252 | 2 | T | C | 0.020 | 0.003 | 2.57E-09 |
| WHR | rs1569135 | 2 | A | G | 0.024 | 0.003 | 1.00E-12 |
| WHR | rs929641 | 2 | A | G | 0.020 | 0.003 | 4.25E-09 |
| WHR | rs17451107 | 3 | T | C | 0.023 | 0.004 | 3.50E-11 |
| WHR | rs9860730 | 3 | A | G | 0.023 | 0.004 | 2.84E-10 |
| WHR | rs459193 | 5 | A | G | 0.026 | 0.004 | 6.02E-12 |
| WHR | rs9491696 | 6 | C | G | -0.038 | 0.003 | 4.88E-30 |
| WHR | rs1358980 | 6 | T | C | 0.027 | 0.004 | 1.98E-14 |
| WHR | rs1294421 | 6 | T | G | -0.026 | 0.003 | 6.93E-14 |
| WHR | rs10245353 | 7 | A | C | 0.027 | 0.004 | 1.57E-10 |
| WHR | rs7801581 | 7 | T | C | 0.023 | 0.004 | 4.95E-08 |
| WHR | rs12549058 | 8 | T | G | -0.040 | 0.006 | 3.17E-10 |
| WHR | rs4929927 | 11 | A | G | -0.020 | 0.003 | 7.60E-09 |
| WHR | rs11048470 | 12 | T | G | 0.025 | 0.004 | 6.33E-12 |
| WHR | rs1443512 | 12 | A | C | 0.026 | 0.004 | 2.76E-11 |
| WHR | rs17109256 | 14 | A | G | 0.023 | 0.004 | 3.02E-08 |
| WHR | rs1440372 | 15 | T | C | -0.021 | 0.004 | 7.59E-09 |
| WHR | rs1121980 | 16 | A | G | 0.043 | 0.003 | 1.33E-38 |
| WHR | rs4640244 | 7 | A | G | -0.021 | 0.004 | 3.11E-08 |
| WHR | rs11663816 | 18 | T | C | -0.025 | 0.004 | 2.65E-11 |
| WHR | rs3786897 | 19 | A | G | -0.022 | 0.003 | 3.95E-11 |
| WHR | rs2075650 | 19 | A | G | 0.029 | 0.005 | 6.43E-09 |
| WHR | rs2287019 | 19 | T | C | -0.026 | 0.005 | 4.34E-09 |
| WHR | rs16996700 | 20 | T | C | 0.021 | 0.004 | 1.60E-08 |
| WHR | rs2179129 | 22 | A | G | 0.021 | 0.003 | 1.24E-09 |
| BMI | rs1558902 | 16 | A | T | 0.082 | 0.003 | 7.50E-153 |
| BMI | rs6567160 | 18 | C | T | 0.056 | 0.004 | 3.93E-53 |
| BMI | rs13021737 | 2 | G | A | 0.060 | 0.004 | 1.11E-50 |
| BMI | rs10938397 | 4 | G | A | 0.040 | 0.003 | 3.20E-38 |
| BMI | rs543874 | 1 | G | A | 0.048 | 0.004 | 2.62E-35 |
| BMI | rs2207139 | 6 | G | A | 0.045 | 0.004 | 4.13E-29 |
| BMI | rs11030104 | 11 | A | G | 0.041 | 0.004 | 5.56E-28 |
| BMI | rs3101336 | 1 | C | T | 0.033 | 0.003 | 2.66E-26 |
| BMI | rs7138803 | 12 | A | G | 0.032 | 0.003 | 8.15E-24 |
| BMI | rs10182181 | 2 | G | A | 0.031 | 0.003 | 8.78E-24 |
| BMI | rs3888190 | 16 | A | C | 0.031 | 0.003 | 3.14E-23 |
| BMI | rs1516725 | 3 | C | T | 0.045 | 0.005 | 1.89E-22 |
| BMI | rs12446632 | 16 | G | A | 0.040 | 0.005 | 1.48E-18 |
| BMI | rs2287019 | 19 | C | T | 0.036 | 0.004 | 4.58E-18 |
| BMI | rs16951275 | 15 | T | C | 0.031 | 0.004 | 1.91E-17 |
| BMI | rs3817334 | 11 | T | C | 0.026 | 0.003 | 5.15E-17 |
| BMI | rs2112347 | 5 | T | G | 0.026 | 0.003 | 6.19E-17 |
| BMI | rs12566985 | 1 | G | A | 0.024 | 0.003 | 3.28E-15 |
| BMI | rs3810291 | 19 | A | G | 0.028 | 0.004 | 4.81E-15 |
| BMI | rs7141420 | 14 | T | C | 0.024 | 0.003 | 1.23E-14 |
| BMI | rs13078960 | 3 | G | T | 0.030 | 0.004 | 1.74E-14 |
| BMI | rs10968576 | 9 | G | A | 0.025 | 0.003 | 6.61E-14 |
| BMI | rs17024393 | 1 | C | T | 0.066 | 0.009 | 7.03E-14 |
| BMI | rs657452 | 1 | A | G | 0.023 | 0.003 | 5.48E-13 |
| BMI | rs12429545 | 13 | A | G | 0.033 | 0.005 | 1.09E-12 |
| BMI | rs12286929 | 11 | G | A | 0.022 | 0.003 | 1.31E-12 |
| BMI | rs13107325 | 4 | T | C | 0.048 | 0.007 | 1.82E-12 |
| BMI | rs11165643 | 1 | T | C | 0.022 | 0.003 | 2.07E-12 |
| BMI | rs7903146 | 10 | C | T | 0.023 | 0.003 | 1.11E-11 |
| BMI | rs10132280 | 14 | C | A | 0.023 | 0.003 | 1.14E-11 |
| BMI | rs17405819 | 8 | T | C | 0.022 | 0.003 | 2.07E-11 |
| BMI | rs1016287 | 2 | T | C | 0.023 | 0.003 | 2.25E-11 |
| BMI | rs4256980 | 11 | G | C | 0.021 | 0.003 | 2.90E-11 |
| BMI | rs17094222 | 10 | C | T | 0.025 | 0.004 | 5.94E-11 |
| BMI | rs12401738 | 1 | A | G | 0.021 | 0.003 | 1.15E-10 |
| BMI | rs7599312 | 2 | G | A | 0.022 | 0.003 | 1.17E-10 |
| BMI | rs2365389 | 3 | C | T | 0.020 | 0.003 | 1.63E-10 |
| BMI | rs205262 | 6 | G | A | 0.022 | 0.004 | 1.75E-10 |
| BMI | rs2820292 | 1 | C | A | 0.020 | 0.003 | 1.83E-10 |
| BMI | rs12885454 | 14 | C | A | 0.021 | 0.003 | 1.94E-10 |
| BMI | rs16851483 | 3 | T | G | 0.048 | 0.008 | 3.55E-10 |
| BMI | rs1167827 | 7 | G | A | 0.020 | 0.003 | 6.33E-10 |
| BMI | rs758747 | 16 | T | C | 0.023 | 0.004 | 7.47E-10 |
| BMI | rs1928295 | 9 | T | C | 0.019 | 0.003 | 7.91E-10 |
| BMI | rs9925964 | 16 | A | G | 0.019 | 0.003 | 8.11E-10 |
| BMI | rs11126666 | 2 | A | G | 0.021 | 0.003 | 1.33E-09 |
| BMI | rs2650492 | 16 | A | G | 0.021 | 0.004 | 1.92E-09 |
| BMI | rs6804842 | 3 | G | A | 0.019 | 0.003 | 2.48E-09 |
| BMI | rs12940622 | 17 | G | A | 0.018 | 0.003 | 2.49E-09 |
| BMI | rs11847697 | 14 | T | C | 0.049 | 0.008 | 3.99E-09 |
| BMI | rs4740619 | 9 | T | C | 0.018 | 0.003 | 4.56E-09 |
| BMI | rs13191362 | 6 | A | G | 0.028 | 0.005 | 7.34E-09 |
| BMI | rs3736485 | 15 | A | G | 0.018 | 0.003 | 7.41E-09 |
| BMI | rs17001654 | 4 | G | C | 0.031 | 0.005 | 7.76E-09 |
| BMI | rs11191560 | 10 | C | T | 0.031 | 0.005 | 8.45E-09 |
| BMI | rs1528435 | 2 | T | C | 0.018 | 0.003 | 1.20E-08 |
| BMI | rs2075650 | 19 | A | G | 0.026 | 0.005 | 1.25E-08 |
| BMI | rs1000940 | 17 | G | A | 0.019 | 0.003 | 1.28E-08 |
| BMI | rs2033529 | 6 | G | A | 0.019 | 0.003 | 1.39E-08 |
| BMI | rs11583200 | 1 | C | T | 0.018 | 0.003 | 1.48E-08 |
| BMI | rs9400239 | 6 | C | T | 0.019 | 0.003 | 1.61E-08 |
| BMI | rs10733682 | 9 | A | G | 0.017 | 0.003 | 1.83E-08 |
| BMI | rs11688816 | 2 | G | A | 0.017 | 0.003 | 1.89E-08 |
| BMI | rs11057405 | 12 | G | A | 0.031 | 0.006 | 2.02E-08 |
| BMI | rs2121279 | 2 | T | C | 0.025 | 0.004 | 2.31E-08 |
| BMI | rs29941 | 19 | G | A | 0.018 | 0.003 | 2.41E-08 |
| BMI | rs11727676 | 4 | T | C | 0.036 | 0.006 | 2.55E-08 |
| BMI | rs3849570 | 3 | A | C | 0.019 | 0.003 | 2.60E-08 |
| BMI | rs6477694 | 9 | C | T | 0.017 | 0.003 | 2.67E-08 |
| BMI | rs7899106 | 10 | G | A | 0.040 | 0.007 | 2.96E-08 |
| BMI | rs2176598 | 11 | T | C | 0.020 | 0.004 | 2.97E-08 |
| BMI | rs2245368 | 7 | C | T | 0.032 | 0.006 | 3.19E-08 |
| BMI | rs17724992 | 19 | A | G | 0.019 | 0.004 | 3.41E-08 |
| BMI | rs7243357 | 18 | T | G | 0.022 | 0.004 | 3.86E-08 |
| BMI | rs1808579 | 18 | C | T | 0.017 | 0.003 | 4.17E-08 |
| BMI | rs2033732 | 8 | C | T | 0.019 | 0.004 | 4.89E-08 |
| UFA | rs10756713 | 9 | A | G | 0.020 | 0.002 | 1.00E-37 |
| UFA | rs10938397 | 4 | A | G | -0.020 | 0.001 | 7.00E-41 |
| UFA | rs11122450 | 1 | T | G | 0.010 | 0.002 | 6.00E-11 |
| UFA | rs11642015 | 16 | C | T | -0.040 | 0.002 | 7.00E-165 |
| UFA | rs11666808 | 19 | T | C | 0.020 | 0.002 | 6.00E-27 |
| UFA | rs13107325 | 4 | C | T | -0.030 | 0.003 | 3.00E-28 |
| UFA | rs1471740 | 3 | T | C | -0.010 | 0.002 | 4.00E-09 |
| UFA | rs17764730 | 5 | C | T | 0.010 | 0.002 | 8.00E-12 |
| UFA | rs2112347 | 5 | T | G | 0.020 | 0.002 | 2.00E-31 |
| UFA | rs2274224 | 10 | G | C | 0.020 | 0.001 | 7.00E-29 |
| UFA | rs236660 | 7 | T | C | -0.010 | 0.002 | 2.00E-19 |
| UFA | rs3764002 | 12 | C | T | 0.020 | 0.002 | 2.00E-29 |
| UFA | rs4776985 | 15 | T | G | 0.020 | 0.002 | 9.00E-31 |
| UFA | rs4790292 | 17 | C | A | 0.020 | 0.002 | 6.00E-24 |
| UFA | rs4876611 | 8 | A | G | -0.020 | 0.002 | 7.00E-28 |
| UFA | rs539515 | 1 | A | C | -0.030 | 0.002 | 9.00E-54 |
| UFA | rs55931203 | 17 | C | T | -0.020 | 0.002 | 6.00E-28 |
| UFA | rs56186137 | 16 | A | G | -0.020 | 0.002 | 3.00E-50 |
| UFA | rs61888762 | 11 | C | G | -0.020 | 0.002 | 3.00E-29 |
| UFA | rs6567160 | 18 | T | C | -0.030 | 0.002 | 5.00E-48 |
| UFA | rs6602997 | 15 | C | T | -0.020 | 0.002 | 6.00E-43 |
| UFA | rs6752378 | 2 | C | A | -0.020 | 0.001 | 7.00E-55 |
| UFA | rs7124681 | 11 | C | A | -0.020 | 0.002 | 7.00E-50 |
| UFA | rs7132908 | 12 | G | A | -0.020 | 0.002 | 1.00E-36 |
| UFA | rs71658797 | 1 | T | A | -0.020 | 0.002 | 6.00E-26 |
| UFA | rs72892910 | 6 | G | T | -0.020 | 0.002 | 2.00E-32 |
| UFA | rs8049669 | 16 | A | T | 0.020 | 0.002 | 7.00E-26 |
| UFA | rs9358912 | 6 | G | T | 0.020 | 0.002 | 5.00E-39 |
| FA | rs10876529 | 12 | T | C | -0.010 | 0.002 | 5.00E-12 |
| FA | rs11045172 | 12 | A | C | -0.010 | 0.002 | 5.00E-09 |
| FA | rs11135038 | 5 | T | G | -0.010 | 0.002 | 3.00E-16 |
| FA | rs113222038 | 11 | C | T | 0.010 | 0.002 | 3.00E-12 |
| FA | rs11664106 | 18 | A | T | -0.010 | 0.002 | 5.00E-09 |
| FA | rs12130231 | 1 | A | G | 0.020 | 0.002 | 4.00E-41 |
| FA | rs12369179 | 12 | C | T | 0.030 | 0.003 | 2.00E-29 |
| FA | rs12441543 | 15 | G | A | -0.010 | 0.002 | 1.00E-12 |
| FA | rs12681990 | 8 | T | C | 0.010 | 0.002 | 5.00E-09 |
| FA | rs12940684 | 17 | C | T | 0.010 | 0.002 | 1.00E-11 |
| FA | rs13132853 | 4 | A | G | 0.010 | 0.002 | 1.00E-08 |
| FA | rs13389219 | 2 | C | T | -0.020 | 0.002 | 2.00E-29 |
| FA | rs142186653 | 17 | A | C | -0.010 | 0.002 | 1.00E-11 |
| FA | rs2802774 | 1 | C | A | -0.010 | 0.002 | 4.00E-13 |
| FA | rs2943653 | 2 | C | T | 0.020 | 0.002 | 1.00E-23 |
| FA | rs2980888 | 8 | T | C | -0.010 | 0.002 | 4.00E-14 |
| FA | rs30351 | 5 | G | A | 0.010 | 0.002 | 2.00E-12 |
| FA | rs4450871 | 4 | A | G | -0.010 | 0.001 | 1.00E-08 |
| FA | rs4684847 | 3 | C | T | -0.030 | 0.002 | 3.00E-37 |
| FA | rs4821764 | 22 | G | A | 0.020 | 0.002 | 3.00E-29 |
| FA | rs4976033 | 5 | A | G | 0.010 | 0.002 | 5.00E-14 |
| FA | rs6029180 | 20 | A | G | -0.010 | 0.002 | 2.00E-08 |
| FA | rs62271373 | 3 | T | A | 0.020 | 0.003 | 2.00E-14 |
| FA | rs6977416 | 7 | G | A | 0.010 | 0.002 | 3.00E-14 |
| FA | rs7133378 | 12 | G | A | -0.020 | 0.002 | 4.00E-33 |
| FA | rs7233512 | 18 | G | A | 0.010 | 0.002 | 8.00E-11 |
| FA | rs7258937 | 19 | C | T | -0.020 | 0.001 | 2.00E-26 |
| FA | rs72697297 | 14 | T | C | 0.020 | 0.002 | 8.00E-15 |
| FA | rs72959041 | 6 | G | A | 0.020 | 0.003 | 2.00E-12 |
| FA | rs972283 | 7 | A | G | 0.010 | 0.001 | 2.00E-22 |
| FA | rs9764678 | 5 | T | C | -0.010 | 0.002 | 3.00E-10 |
| FA | rs9851766 | 3 | A | G | 0.010 | 0.002 | 1.00E-11 |
| FA | rs987469 | 4 | C | G | 0.010 | 0.001 | 6.00E-17 |
| FA | rs998584 | 6 | C | A | 0.010 | 0.001 | 1.00E-09 |
| RT | rs10125715 | 9 | T | A | 0.010 | 0.002 | 1.47E-08 |
| RT | rs1032501 | 3 | C | G | 0.010 | 0.002 | 1.11E-08 |
| RT | rs1054442 | 12 | C | A | 0.012 | 0.002 | 2.44E-12 |
| RT | rs10804171 | 2 | C | T | 0.010 | 0.002 | 4.25E-08 |
| RT | rs10911301 | 1 | A | G | 0.010 | 0.002 | 1.83E-09 |
| RT | rs11205668 | 1 | T | C | 0.010 | 0.002 | 2.68E-08 |
| RT | rs13219424 | 6 | C | T | 0.010 | 0.002 | 1.33E-08 |
| RT | rs1351848 | 11 | T | C | 0.011 | 0.002 | 9.48E-10 |
| RT | rs1385253 | 4 | T | C | 0.010 | 0.002 | 1.67E-08 |
| RT | rs1503043 | 17 | G | C | 0.010 | 0.002 | 2E-08 |
| RT | rs16822665 | 2 | C | T | 0.010 | 0.002 | 4.94E-09 |
| RT | rs16959783 | 15 | T | C | 0.010 | 0.002 | 1.8E-08 |
| RT | rs1734197 | 16 | T | C | 0.010 | 0.002 | 2.2E-08 |
| RT | rs2040879 | 7 | C | T | 0.011 | 0.002 | 3.31E-10 |
| RT | rs2604268 | 9 | T | C | 0.012 | 0.002 | 9.2E-12 |
| RT | rs264979 | 2 | T | A | 0.013 | 0.002 | 5.93E-13 |
| RT | rs2869529 | 2 | T | C | 0.010 | 0.002 | 1.89E-09 |
| RT | rs323299 | 18 | G | T | 0.010 | 0.002 | 1.34E-08 |
| RT | rs35066740 | 2 | T | G | 0.010 | 0.002 | 3.68E-09 |
| RT | rs4627212 | 13 | G | A | 0.010 | 0.002 | 1.88E-09 |
| RT | rs4673905 | 2 | G | A | 0.012 | 0.002 | 3.98E-12 |
| RT | rs4852778 | 2 | G | A | 0.010 | 0.002 | 3.75E-08 |
| RT | rs4946935 | 6 | A | G | 0.012 | 0.002 | 7.69E-12 |
| RT | rs56335290 | 5 | A | C | 0.012 | 0.002 | 1.97E-12 |
| RT | rs58984824 | 12 | A | C | 0.010 | 0.002 | 2.18E-08 |
| RT | rs61786416 | 1 | G | A | 0.010 | 0.002 | 1.66E-08 |
| RT | rs62074125 | 17 | C | A | 0.012 | 0.002 | 5.28E-12 |
| RT | rs648997 | 12 | C | T | 0.013 | 0.002 | 2.07E-12 |
| RT | rs66534382 | 19 | G | A | 0.011 | 0.002 | 1.64E-10 |
| RT | rs6870103 | 5 | T | G | 0.010 | 0.002 | 1.88E-09 |
| RT | rs7075591 | 10 | A | G | 0.010 | 0.002 | 1.33E-08 |
| RT | rs73464507 | 11 | G | C | 0.011 | 0.002 | 1.05E-09 |
| RT | rs75543711 | 17 | G | A | 0.010 | 0.002 | 4.6E-08 |
| RT | rs7598246 | 2 | C | T | 0.010 | 0.002 | 3.61E-08 |
| RT | rs7783359 | 7 | A | T | 0.010 | 0.002 | 2.69E-08 |
| RT | rs77917390 | 5 | T | A | 0.010 | 0.002 | 1.68E-08 |
| RT | rs77998199 | 2 | G | A | 0.011 | 0.002 | 1.97E-10 |
| RT | rs7936413 | 11 | C | T | 0.011 | 0.002 | 2.11E-09 |
| RT | rs80054135 | 7 | A | T | 0.010 | 0.002 | 2.34E-08 |
| RT | rs925229 | 2 | G | A | 0.010 | 0.002 | 4.66E-08 |
| RT | rs936414 | 12 | G | A | 0.010 | 0.002 | 4.95E-08 |
| VM | rs1007876 | 22 | G | A | 0.027 | 0.005 | 1.29E-08 |
| VM | rs10105797 | 8 | C | T | -0.026 | 0.004 | 2.94E-09 |
| VM | rs11191193 | 10 | G | A | 0.027 | 0.004 | 1.22E-09 |
| VM | rs11202929 | 10 | G | A | 0.024 | 0.004 | 5.63E-09 |
| VM | rs11215690 | 11 | G | A | -0.026 | 0.004 | 1.03E-09 |
| VM | rs1319892 | 12 | C | T | 0.025 | 0.004 | 6.1E-09 |
| VM | rs13337187 | 16 | C | T | -0.038 | 0.006 | 1.03E-09 |
| VM | rs180204 | 8 | T | C | 0.026 | 0.005 | 2.18E-08 |
| VM | rs1927551 | 13 | G | A | -0.034 | 0.006 | 3.86E-09 |
| VM | rs1934827 | 13 | A | T | -0.026 | 0.004 | 8.67E-10 |
| VM | rs2195450 | 5 | A | G | 0.030 | 0.005 | 1.41E-09 |
| VM | rs224768 | 10 | A | G | 0.025 | 0.005 | 3.62E-08 |
| VM | rs2273709 | 6 | C | A | -0.030 | 0.005 | 6.73E-09 |
| VM | rs2453762 | 5 | A | G | -0.026 | 0.005 | 1.37E-08 |
| VM | rs28374800 | 16 | G | C | -0.028 | 0.005 | 2.48E-08 |
| VM | rs2862954 | 10 | C | T | -0.023 | 0.004 | 3.65E-08 |
| VM | rs2953617 | 7 | A | T | -0.025 | 0.004 | 5.49E-09 |
| VM | rs35256954 | 7 | A | G | 0.031 | 0.006 | 3.43E-08 |
| VM | rs35408576 | 5 | C | A | -0.029 | 0.005 | 1.82E-09 |
| VM | rs4360998 | 18 | T | C | -0.024 | 0.004 | 1.06E-08 |
| VM | rs4420638 | 19 | G | A | 0.031 | 0.005 | 3.77E-09 |
| VM | rs4889606 | 16 | G | A | 0.028 | 0.004 | 1.1E-10 |
| VM | rs55732507 | 16 | C | T | 0.032 | 0.004 | 1.22E-13 |
| VM | rs6995556 | 8 | T | C | -0.031 | 0.004 | 4.3E-12 |
| VM | rs7316448 | 12 | C | T | -0.032 | 0.005 | 4.08E-11 |
| VM | rs749694 | 10 | G | A | 0.026 | 0.004 | 7.81E-10 |
| VM | rs7582485 | 2 | G | C | 0.032 | 0.004 | 5E-14 |
| VM | rs79494112 | 6 | C | T | 0.070 | 0.013 | 4.95E-08 |
| VM | rs9295740 | 6 | A | G | -0.030 | 0.005 | 1.36E-08 |
| VM | rs9319606 | 17 | G | T | 0.025 | 0.004 | 7.86E-09 |

*Where necessary, beta coefficients were multiplied by -1 to ensure all betas represented an increase in the respective traits and allele harmonisation was done to ensure alignment of alleles for both the single nucleotide polymorphism(SNP)-X and SNP-Y associations; GWAS betas, standard errors (SEs) and *P*-values taken from Locke et al. 2015. Nature, 518(7538):197-206 (body mass index (BMI)); Shungin et al. 2015. Nature, 518(7538):187-96 (waist-hip ratio (WHR)); Lu et al. 2016. Nature comm, 7(1):1-15 (body fat percentage (BF%)); Martin et al. 2021. Diabetes, 70(8): 1843-1856 (unfavourable/favourable adiposity (UFA/FA)); Davies et al. 2018. Nature comms, 9(1):1-16 (Reaction time (RT)); Neale lab- UK Biobank 2022 [Available from: <http://www.nealelab.is/uk-biobank>] (Visual memory (VM)).

## **Supplementary Table S4: Details of genetic instruments used**

| ***Adiposity to cognition*** | | | | | |
| --- | --- | --- | --- | --- | --- |
| Adiposity instrument | GWAS | Number SNPs | F-statistic | R^2^ | Sample overlap (with UKB)? |
| BF% | Lu et al. 2016 | 6 | 53.44 | 0.08% | No |
| WHR | Shungin et al. 2015 | 28 | 52.11 | 0.38% | No |
| BMI | Locke et al. 2015 | 76 | 91.43 | 1.80% | No |
| ‘Unfavourable’ adiposity | Martin et al. 2021 | 28 | 64.48 | 0.47% | Yes |
| ‘Favourable’ adiposity | Martin et al. 2021 | 34 | 24.84 | 0.22% | Yes |
| ***Cognition to adiposity*** | | | | | |
| Cognition instrument | GWAS | Number SNPs | F-statistic | R^2^ | Sample overlap (with UKB)? |
| RT | Davies et al. 2018 | 41 | 32.64 | 0.35% | Yes |
| VM | Neale lab (public) | 30 | 22.01 | 0.17% | Yes |

GWAS=genome wide association study; BF%=ody fat percentage; WHR=Waist-hip ratio; BMI=Body mass index; RT=Reaction time; VM=Visual memory; SNP-single nucleotide polymorphism; UKB=UK Biobank

## **Supplementary table S5: Removing adiposity SNPs associated with potential confounders**

| **Percentage difference (95% CI, *P*-value) in reaction time by adiposity indicators** | | | | | |
| --- | --- | --- | --- | --- | --- |
| MR analyses: removing SNPs associated with potential confounders | | | | | |
|  | BF% | BMI | WHR | UFA | FA |
| *Number of SNPs removed* | 3 | 33 | 11 | 19 | 5 |
| IVW | -2.26 (-4.24, -0.24, 0.03) | -0.98 (-1.82, -0.12, 0.03) | 0.04 (-0.85, 0.94, 0.93) | -3.53 (-5.94, -1.06, 0.001) | -0.35 (-2.22, 1.55, 0.71) |
| I^2^ | 0.38 | 0.52 | 0.03 | 0.52 | 0.58 |
| WME | -1.55 (-3.55, 0.49, 0.14) | -1.17 (-2.03, -0.29, 0.01) | 0.36 (-0.89, 1.61, 0.58) | -3.83 (-6.25, -1.34, 0.003) | -1.38 (-3.29, 0.56, 0.16) |
| MR-Egger | -19.82 (-35.46, -0.39, 0.05) | -0.43 (-2.99, 2.20, 0.75) | 2.17 (-2.56, 7.14, 0.38) | 0.22 (-8.94, 10.31, 0.96) | 1.37 (-3.66, 6.66, 0.60) |
| *P-*pleiotropy | 0.07 | 0.66 | 0.38 | 0.42 | 0.48 |
| I^2^_GX_ | - | 0.67 | 0.49 | 0.90 | 0.88 |
| **Percentage difference (95% CI, *P*-value) in visual memory by adiposity indicators** | | | | | |
| MR analyses: removing SNPs associated with potential confounders | | | | | |
|  | BF% | BMI | WHR | UFA | FA |
| *Number of SNPs removed* | 3 | 33 | 11 | 19 | 5 |
| IVW | 6.03 (-1.58, 14.24, 0.12) | -0.07 (-3.11, 3.06, 0.96) | 0.58 (-2.86, 4.14, 0.75) | -12.40 (-18.32, -6.05, <0.001) | 1.23 (-4.85, 7.70, 0.70) |
| I^2^ | 0.44 | 0.56 | 0.23 | 0.26 | 0.53 |
| WME | 7.11 (-0.88, 15.75, 0.08) | 0.21 (-3.19, 3.73, 0.90) | 1.37 (-3.05, 6.00, 0.55) | -10.50 (-17.81, -2.53, 0.01) | 0.43 (-6.70, 8.11, 0.91) |
| MR-Egger | 16.01 (-71.56, 373.22, 0.84) | -1.25 (-10.09, 8.46, 0.79) | -20.89 (-32.59, -7.17, 0.004) | -8.25 (-30.32, 20.80, 0.54) | 2.65 (-13.25, 21.46, 0.76) |
| *P*-pleiotropy | 0.90 | 0.79 | 0.003 | 0.73 | 0.86 |
| I^2^_GX_ | - | 0.67 | 0.49 | 0.90 | 0.88 |

IVW: Inverse-variance-weighted; WME: Weighted median estimator; MR-Egger: Mendelian randomisation Egger regression; BF%=Body fat percentage; WHR=Waist-hip ratio; BMI=Body mass index; UFA=unfavourable adiposity; FA=favourable adiposity; SNP=single nucleotide polymorphism

## **Supplementary table S6: Removing cognitive function SNPs associated with potential confounders**

|  | **Difference (95% CI, *P*-value) in BF% by cognitive function** | |
| --- | --- | --- |
|  | RT | VM |
|  | MR analyses: removing SNPs associated with potential confounders | |
| *Number of SNPs removed* | 8 | 11 |
| IVW | 0.55 (-0.91, 2.00, 0.46) | -0.89 (-1.36, -0.42, <0.001) |
| I^2^ | 0.74 | 0.44 |
| WME | -0.11 (-1.30, 1.08, 0.86) | -0.91 (-1.45, -0.38, 0.001) |
| MR-Egger | 7.09 (-13.77, 27.96, 0.51) | -1.42 (-1.53, 4.36, 0.35) |
| *P-*pleiotropy | 0.54 | 0.72 |
| I^2^_GX_ | 0.45 | 0.00 |
|  | **Difference (95% CI, *P*-value) in WHR by cognitive function** | |
|  | RT | VM |
|  | MR analyses: removing SNPs associated with potential confounders | |
| *Number of SNPs removed* | 8 | 11 |
| IVW | 0.01 (-0.001, 0.02, 0.09) | 0.001 (-0.01, 0.01, 0.68) |
| I^2^ | 0.43 | 0.64 |
| WME | 0.01 (0.002, 0.03, 0.03) | -0.0004 (-0.01, 0.01, 0.89) |
| MR-Egger | 0.14 (-0.005, 0.28, 0.06) | 0.01 (-0.03, 0.05, 0.55) |
| *P*-pleiotropy | 0.08 | 0.59 |
| I^2^_GX_ | 0.45 | 0.00 |
|  | **Percent difference (95% CI, *P*-value) in BMI by cognitive function** | |
|  | RT | VM |
|  | MR analyses: removing SNPs associated with potential confounders | |
| *Number of SNPs removed* | 8 | 11 |
| IVW | -2.72 (-0.99, 6.56, 0.15) | -2.12 (-3.68, -0.58, 0.01) |
| I^2^ | 0.85 | 0.80 |
| WME | 0.17 (-2.62, 3.03, 0.91) | -1.83 (-3.11, -0.55, 0.01) |
| MR-Egger | 61.32 (-2.67, 167.39, 0.06) | -9.20 (-19.41, 0.13, 0.05) |
| *P*-pleiotropy | 0.08 | 0.14 |
| I^2^_GX_ | 0.45 | 0.00 |

IVW: Inverse-variance-weighted; WME: weighted median estimator; MR-Egger: Mendelian randomisation Egger regression; BF%=Body fat percentage; WHR=Waist-hip ratio; BMI=Body mass index; RT=Reaction time; VM=Visual memory; SNP=single nucleotide polymorphism

## **Supplementary Table S7: Removing potentially pleiotropic SNPs from analysis of associations between WHR and VM**

| **Percentage difference (95% CI, *P*-value) in VM** | |
| --- | --- |
| **Removing potentially pleiotropic SNPs** | |
|  |  |
| *Number of SNPs removed* | 4 |
| IVW | 1.63 (-1.32, 4.67, 0.28) |
| I^2^ | 0.14 |
| WME | 1.50 (-2.49, 5.66, 0.47) |
| MR-Egger | -8.97 (-30.32, 18.93, 0.49) |
| *P-*pleiotropy | 0.42 |
| I^2^_GX_ | 0.00 |
| **Removing potentially pleiotropic SNPs and SNPs associated with potential confounders** | |
|  |  |
|  |  |
| *Number of SNPs removed* | 15 (4+11) |
| IVW | 3.38 (-0.08, 6.97, 0.06) |
| I^2^ | 0.00 |
| WME | 2.51 (-2.01, 7.25, 0.28) |
| MR-Egger | 3.37 (-24.93, 42.35, 0.84) |
| *P*-pleiotropy | 1.00 |
| I^2^_GX_ | 0.00 |

IVW: Inverse-variance-weighted; WME: weighted median estimator; MR-Egger: Mendelian randomisation Egger regression; WHR=Waist-hip ratio; VM=Visual memory; SNP-single nucleotide polymorphism

## **Supplementary Table S8: Estimation of bias due to sample overlap**

| **Exposure** | **Outcome** | **OLS estimate*** | **N**  **(outcome)** | **Number of exposure SNPs** | **R^2^**  **(for exposure IV)** | **F-statistic**  **(for exposure IV)** | **N (instrument)** | **Bias**  **(100% overlap)***** | **Type-1 error rate** |
| --- | --- | --- | --- | --- | --- | --- | --- | --- | --- |
| BF% | RT | 0.1091978 | 378877 | 6 | 0.0008 | 53.44 | 378877 | 0.002043372 | 0.05 |
|  | VM | 0.0091905 | 378877 | 6 | 0.0008 | 53.44 | 378877 | 0.000171978 | 0.05 |
| BMI | RT | 0.0120948 | 378877 | 76 | 0.018 | 91.43 | 378877 | 0.000132285 | 0.05 |
|  | VM | -0.0202367 | 378877 | 76 | 0.018 | 91.43 | 378877 | -0.000221335 | 0.05 |
| WHR | RT | 0.0109923 | 378877 | 28 | 0.0038 | 52.11 | 378877 | 0.000210944 | 0.05 |
|  | VM | 0.0080204 | 378877 | 28 | 0.0038 | 52.11 | 378877 | 0.000153913 | 0.05 |
| UFA** | RT | 0.1091978 | 378877 | 28 | 0.0047 | 64.48 | 378877 | 0.001693514 | 0.05 |
|  | VM | 0.0091905 | 378877 | 28 | 0.0047 | 64.48 | 378877 | 0.000142533 | 0.05 |
| FA** | RT | 0.1091978 | 378877 | 34 | 0.0022 | 24.84 | 378877 | 0.004396047 | 0.05 |
|  | VM | 0.0091905 | 378877 | 34 | 0.0022 | 24.84 | 378877 | 0.000369988 | 0.05 |
| RT | BF% | 0.0994626 | 378877 | 41 | 0.0035 | 32.64 | 378877 | 0.003047261 | 0.05 |
|  | BMI | 0.0101564 | 378877 | 41 | 0.0035 | 32.64 | 378877 | 0.000311164 | 0.05 |
|  | WHR | 0.0129453 | 378877 | 41 | 0.0035 | 32.64 | 378877 | 0.000396608 | 0.05 |
| VM | BF% | -0.001909 | 378877 | 30 | 0.0017 | 22.01 | 378877 | -8.67333E-05 | 0.05 |
|  | BMI | -0.021858 | 378877 | 30 | 0.0017 | 22.01 | 378877 | -0.000993094 | 0.05 |
|  | WHR | 0.0149778 | 378877 | 30 | 0.0017 | 22.01 | 378877 | 0.0006805 | 0.05 |

*Ordinary least square (OLS) regression estimates differ to those shown in Tables 2 and 3 as outcomes are in standard deviation units (e.g., outcome = reaction time/SD(reaction time); var(outcome)=1). Numbers represent the association between a SD increase in the respective exposure traits and a SD of the respective outcomes. **Bias approximated using ordinary least squares (OLS) estimates obtained from regressions with BF% as no UFA/FA phenotype measured in UKB. ***Estimated bias assuming 100% sample overlap. BF%=Body fat percentage; WHR=Waist-hip ratio; BMI=Body mass index; RT=Reaction time; VM=Visual memory; SNP-single nucleotide polymorphism

Bias and type-1 error rate were calculated following formula from: *Burgess S, Davies NM, Thompson SG. Bias due to participant overlap in two-sample Mendelian randomization. Genet Epidemiol. 2016;40(7):597-608.*

##

## **Supplementary Table S9. Causal effects of cognitive function on adiposity: split sample strategy***

|  | **Difference (95% CI, *P*-value) in BF% by cognitive function** | |
| --- | --- | --- |
|  | RT | VM |
|  | MR analyses | |
| *Number of SNPs* | 41 | 30 |
| IVW | -0.002 (-0.01, 0.01, 0.68) | -0.79 (-1.06, -0.51, <0.001) |
| WME | -0.001 (-0.01, 0.01, 0.78) | -0.77 (-1.05, -0.49, <0.001) |
| MR-Egger | -0.002 (-0.04, 0.04, 0.91) | -0.26 (-1.26, 0.74, 0.61) |
|  | **Difference (95% CI, *P*-value) in WHR by cognitive function** | |
|  | RT | VM |
|  | MR analyses | |
| *Number of SNPs* | 41 | 30 |
| IVW | 1*10^-6^ (-1*10^-4^, 1*10^-4^, 0.96) | -0.003 (-0.01, 0.0001, 0.06) |
| WME | 3*10^-5^ (-4*10^-4^, 1*10^-4^, 0.47) | -3*10^-4^ (-0.003, 0.003, 0.83) |
| MR-Egger | 1*10^-4^ (-1*10^-4^, 2*10^-4^, 0.62) | -0.002 (-0.0.1, 0.01, 0.74) |
|  | **Percent difference (95% CI, *P*-value) in BMI by cognitive function** | |
|  | RT | VM |
|  | MR analyses | |
| *Number of SNPs* | 41 | 30 |
| IVW | -0.002 (-0.02, 0.02, 0.82) | -2.18 (-3.15, -1.44, <0.001) |
| WME | -0.01 (-0.03, 0.003, 0.14) | -1.61 (-2.21, -1.00, <0.001) |
| MR-Egger | -1*10^-4^ (-0.11, 0.11, 0.99) | -2.98 (-5.73, -0.15, 0.04) |

IVW: Inverse-variance-weighted; WME: weighted median estimator; MR-Egger: Mendelian randomisation Egger regression; BF%=Body fat percentage; WHR=Waist-hip ratio; BMI=Body mass index; RT=Reaction time; VM=Visual memory; SNP-single nucleotide polymorphism *Estimates obtained from the random effects meta-analysis of the estimates obtained using: 1) the MR of SNP-X from sample A and SNP-Y from sample B (*A on B*) and 2) vice versa (*B on A*); see methods for details.

# **Supplementary Figures**

## **Supplementary Figure S1: Adiposity-cognitive function directed acyclic graph (DAG)***


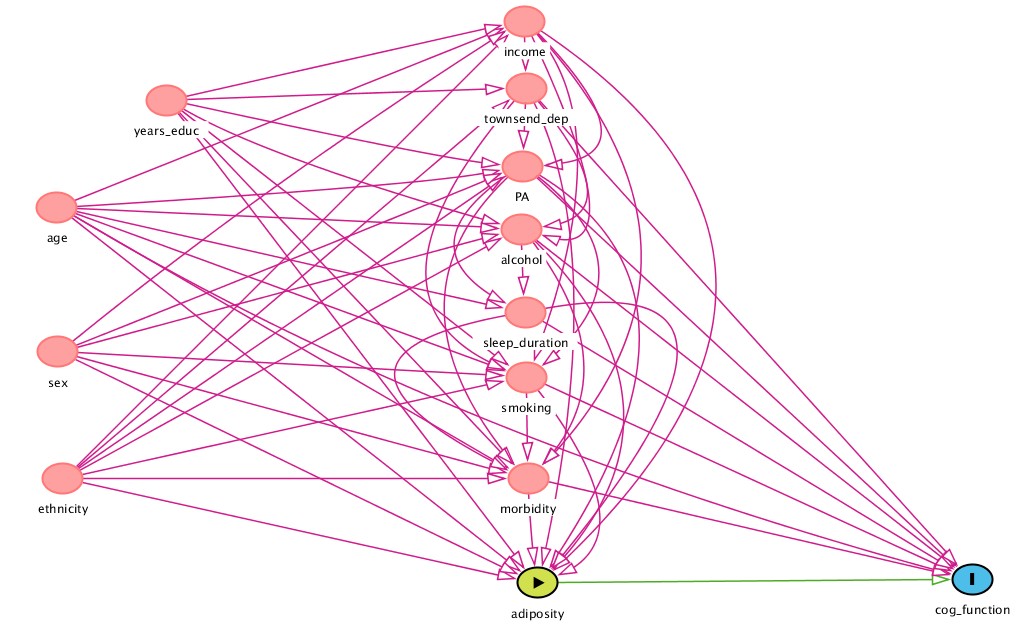


*constructed using the online tool DAGitty (<https://www.dagitty.net>)

## **Supplementary Figure S2: Cognitive function-adiposity directed acyclic graph (DAG)***


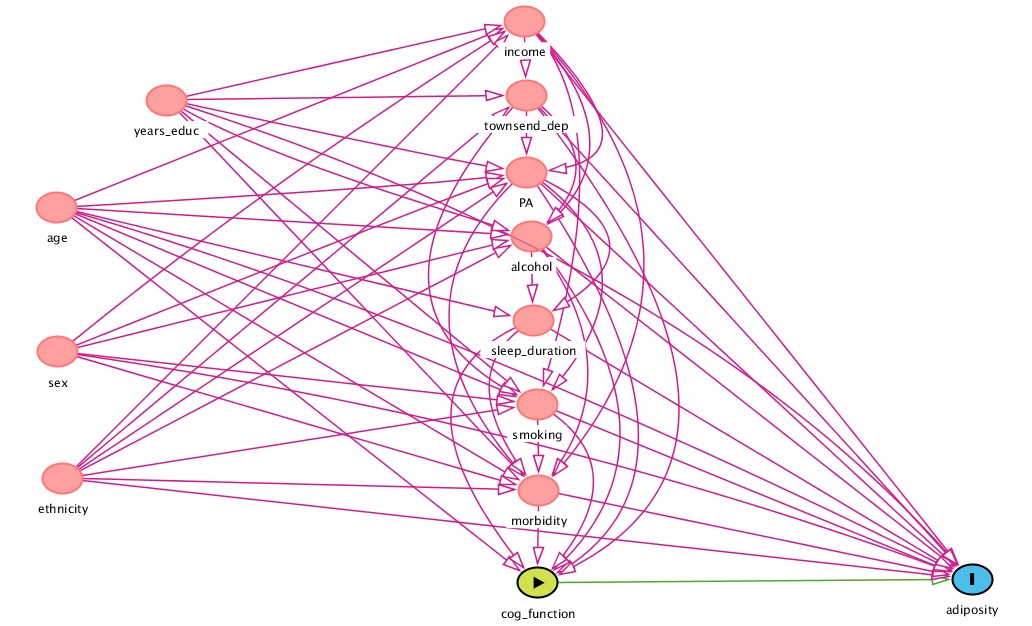


*constructed using the online tool DAGitty (<https://www.dagitty.net>)

## **Supplementary Figure S3: Observational and MR estimates of association between adiposity and reaction time**


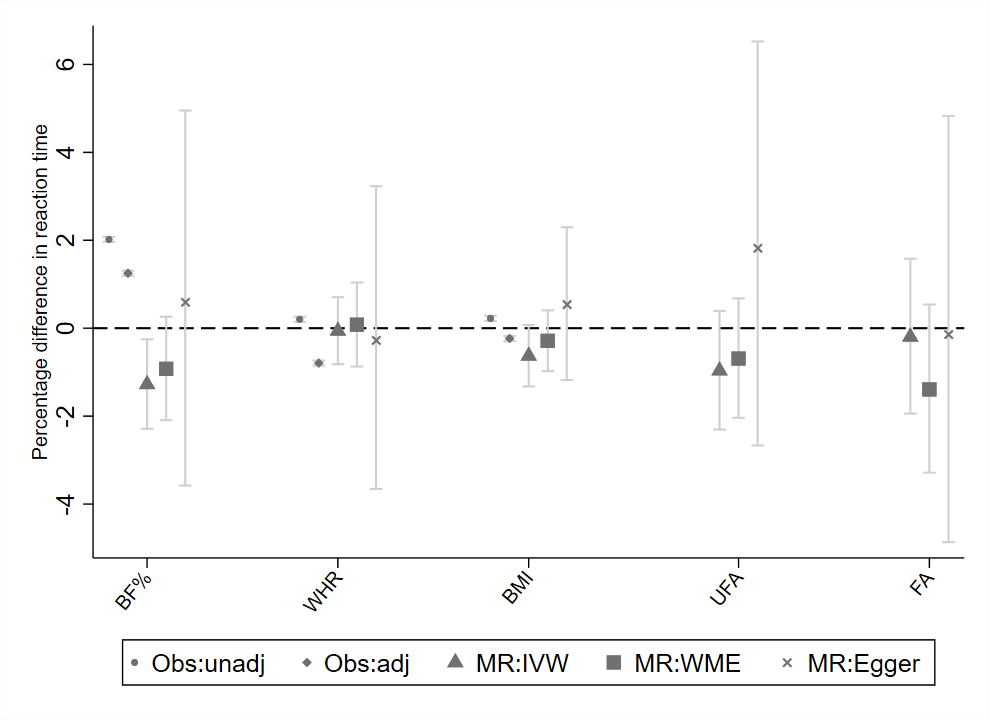


Obs:unaj= unadjusted observational estimates; Obs-adj= adjusted observational estimates; MR-IVW: Inverse-variance-weighted; MR-WME: weighted median estimator; MR-Egger: Mendelian randomisation Egger regression; BF%=Body fat percentage; WHR=Waist-hip ratio; BMI=Body mass index; UFA=Unfavourable adiposity; FA=Favourable adiposity

## **Supplementary Figure S4: Observational and MR estimates of association between adiposity and visual memory**


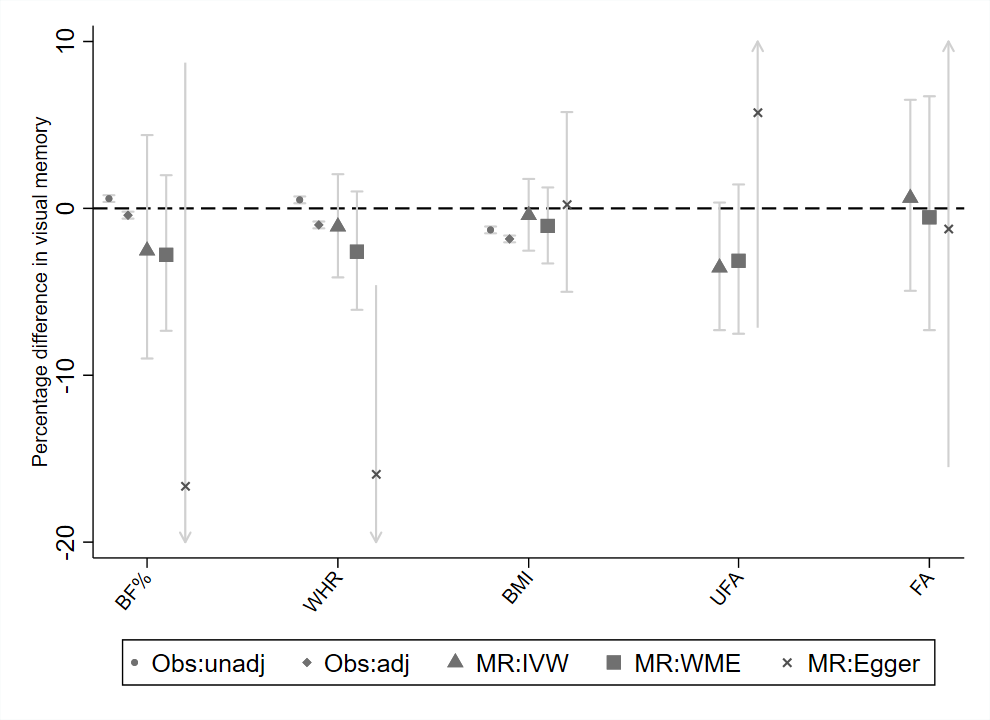


Obs:unaj= unadjusted observational estimates; Obs:adj= adjusted observational estimates; MR-IVW: Inverse-variance-weighted; MR-WME: weighted median estimator; MR-Egger: Mendelian randomisation Egger regression; BF%=Body fat percentage; WHR=Waist-hip ratio; BMI=Body mass index; UFA=Unfavourable adiposity; FA=Favourable adiposity

## **Supplementary Figure S5: Observational and MR estimates of association between cognitive function and body fat percentage**


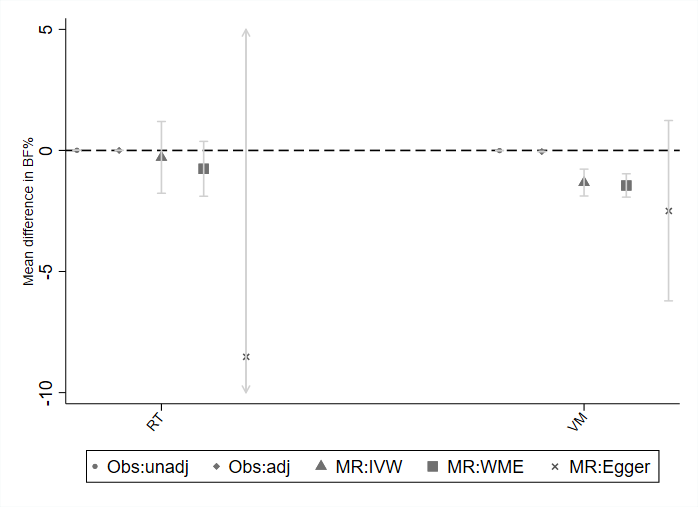


Obs:unad=unadjusted observational estimates; Obs:adj= adjusted observational estimates; MR-IVW: Inverse-variance-weighted; MR-WME: weighted median estimator; MR-Egger: Mendelian randomisation Egger regression; BF%=Body fat percentage; RT=Reaction time; VM=Visual memory

## **Supplementary Figure S6: Observational and MR estimates of association between cognitive function and waist-hip ratio**


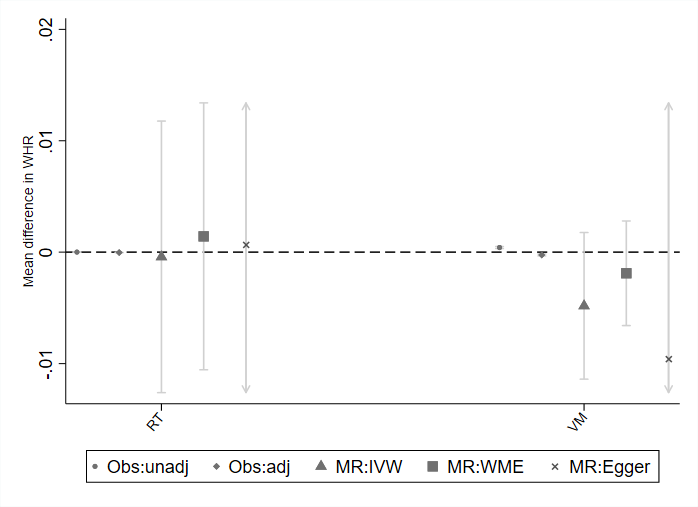


Obs:unad=unadjusted observational estimates; Obs:adj= adjusted observational estimates; MR-IVW: Inverse-variance-weighted; MR-WME: weighted median estimator; MR-Egger: Mendelian randomisation Egger regression; WHR=Waist-hip ratio; RT=Reaction time; VM=Visual memory

## **Supplementary Figure S7: Observational and MR estimates of association between cognitive function and body mass index**


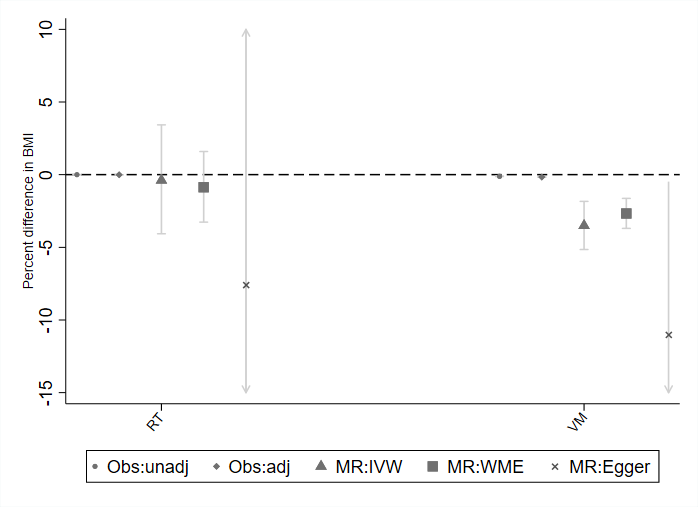


Obs:unad=unadjusted observational estimates; Obs:adj= adjusted observational estimates; MR-IVW: Inverse-variance-weighted; MR-WME: weighted median estimator; MR-Egger: Mendelian randomisation Egger regression; BMI=Body mass index; RT=Reaction time; VM=Visual memory

# **References**

1. Bycroft C, Freeman C, Petkova D, et al. The UK Biobank resource with deep phenotyping and genomic data. Nature. 2018;562(7726):203-9.

2. Davey Smith G, Hemani G. Mendelian randomization: genetic anchors for causal inference in epidemiological studies. Hum Mol Genet. 2014;23(R1):R89-R98.

3. Burgess S, Bowden J. Integrating summarized data from multiple genetic variants in Mendelian randomization: bias and coverage properties of inverse-variance weighted methods. arXiv, doi:<https://arxiv.org/abs/1512.04486v1>, 27 November 2015, preprint: not peer reviewed.

4. Burgess S, Bowden J, Fall T, Ingelsson E, Thompson SG. Sensitivity analyses for robust causal inference from Mendelian randomization analyses with multiple genetic variants. Epidemiology. 2017;28(1):30.

5. Bowden J, Del Greco M F, Minelli C, Davey Smith G, Sheehan N, Thompson J. A framework for the investigation of pleiotropy in two‐sample summary data Mendelian randomization. Stat Med. 2017;36(11):1783-802.

6. Bowden J, Davey Smith G, Haycock PC, Burgess S. Consistent estimation in Mendelian randomization with some invalid instruments using a weighted median estimator. Genet Epidemiol. 2016;40(4):304-14.

7. Burgess S, Bowden J, Dudbridge F, Thompson SG. Robust instrumental variable methods using multiple candidate instruments with application to Mendelian randomization. arXiv, doi:<https://arxiv.org/abs/1606.03729>, 12 June 2016, preprint: not peer reviewed
